# In-Vivo 6D Heart Motion Analysis for Emerging Self-Powered Cardiac Implants

**DOI:** 10.1007/s10439-026-04037-5

**Published:** 2026-02-19

**Authors:** Milad Hasani, John Huber, Benedict Kjærgaard, Tomas Zaremba, Alireza Rezania, Sam Riahi

**Affiliations:** 1 Department of Energy, Aalborg University, Aalborg, Denmark; 2 Department of Engineering Science, University of Oxford, Oxford, United Kingdom; 3 Department of Clinical Medicine, Aalborg University, Aalborg, Denmark; 4 Department of Cardiothoracic Surgery, Aalborg University Hospital, Aalborg, Denmark; 5 Department of Cardiology, Aalborg University Hospital, Aalborg, Denmark

**Keywords:** Cardiac motion, Self-powered implants, Piezoelectric energy harvesting, Intracardiac device, Inertial measurement, In vivo porcine model

## Abstract

Self-powered intracardiac implant devices show great promise for future clinical applications due to their extended operational lifespan and the potential to reduce the need for high-risk repeat surgeries. This study investigates the feasibility of harvesting energy from cardiac motion through in vivo testing of intracardiac devices. Comprehensive three-dimensional translational and rotational cardiac motions are captured in a porcine model using a miniaturized 9-degree-of-freedom motion sensor implanted at six strategic epicardial sites. Kinematic criteria are developed to evaluate the energy harvesting potential of each implant site based on the available kinetic energy, acceleration, and jerk factors. The recorded heart motion signals are analyzed and applied to a conceptual energy harvester proposed to identify the optimal implant site. The results reveal that the left ventricular apex emerges as a preferable site for energy harvesting, particularly at moderate heart rates. These findings offer valuable insights into optimizing self-powered intracardiac implants, reducing dependency on battery replacements, and enhancing long-term patient safety.

**Supplementary Information:**

The online version contains supplementary material available at 10.1007/s10439-026-04037-5.

## Introduction

Intracardiac implant devices enable a broad spectrum of functionalities crucial for cardiovascular health, ranging from precise electrical pacing and life-saving defibrillation to comprehensive diagnostic monitoring and advanced therapeutic applications. Intracardiac leadless pacemakers (ICLPs) have revolutionized cardiac pacing by eliminating the risks associated with traditional lead-based pacemakers, though early models were limited to single-chamber pacing. Advances in wireless synchronization now enable multi-chamber pacing, expanding their potential as a safer and less invasive alternative for a broader range of patients. The ICLPs rely on miniature integrated batteries with limited energy capacity. Replacing a pacemaker device (when the battery is depleted) needs surgical procedures that carry inherent risks and can be life-threatening for patients [1]. To address this limitation, researchers are exploring alternative energy sources that provide a continuous power supply and eliminate the need for high-risk replacement procedures. Energy harvesting from ambient environmental sources is a promising approach for this application. For instance, the kinetic energy of the human body and heart has been investigated as a power source for implantable cardiac devices, especially ICLPs [2, 3].

Recent advances in energy harvesting have explored various mechanisms to eliminate battery replacement and extend intracardiac device longevity [4]. Electromagnetic induction-based harvesters leverage heart motion to drive a mass imbalance oscillation generator or oscillating magnet [5, 6]. These designs generated up to 80 μW, sufficient for pacemaker function but requiring careful placement for optimal performance. The electromagnetic energy harvesters have been improved by a compact design for leadless cardiac pacemakers [7, 8], consisting of a miniaturized oscillating magnet system that converts heart motion into electrical energy via electromagnetic induction. Unlike larger harvesters, these miniature designs are optimized for implantation inside leadless pacemakers. Moreover, a vascular turbine system has been explored [9], which harnesses blood flow to generate power. While this offers a continuous energy supply, thrombus formation and long-term biocompatibility remain concerns. Similarly, an intracardiac flow-based electromagnetic harvester [10] has been investigated to utilize blood flow in the right ventricular outflow tract, producing up to 82.64 μW, but requiring further in vivo validation.

Unlike electromagnetic energy harvesters, which contain permanent magnetic elements that interact with MRI scanning's magnetic field, piezoelectric energy harvesters offer better MRI compatibility because their materials are mainly unaffected by magnetic fields. An early study [11] on piezoelectric energy harvesting for pacemakers investigated a fan-folded intracardiac device, predicting its performance from heart motion. However, the experimental prototype was considerably larger than the proposed miniature capsule. Moreover, the long-term in vivo performance of an innovative hexa-fold piezoelectric energy harvester has recently been investigated for self-powered leadless pacemakers [12]. In another study [2], a PEH based on multiple spiral piezoelectric beams is investigated under one-dimensional motion normal to the epicardium (the outermost layer of the heart) measured at different sites by a laser sensor. The results have shown that the motion of the implant site can considerably affect the harvested energy level.

The heart muscle, or myocardium, contains fibers arranged in a helical pattern, causing the heart to twist during each contraction [13]. This twisting motion, known as ventricular torsion, enhances the efficiency of blood ejection. Therefore, beyond 3D translational movements, the twisting/rotational cardiac motion should be considered in the design of endocardial energy harvesters. However, previous research mainly focused on deriving 1D and 3D translational cardiac motions via MRI scanning [14], accelerometers [15–17], 1D laser measurements [2], image processing [18, 19], echocardiography [20], and biplane videofluoroscopy and radiopaque beads [21]. The rotational motion measurement has been unmet in the previous studies.

This study aims to fill a gap in the literature by completely characterizing both 3D translational and rotational cardiac motion patterns at various epicardial sites and heart rates. In this regard, a 9-degree-of-freedom (DOF) motion sensor is implanted in six predefined epicardial points of a living pig heart to measure 3D translational and rotational movements at various heart rates. We use the measured heart motion to identify optimal implant sites and to guide energy-harvester design. Since evaluating the energy harvesting performance of each implant site depends on the energy harvester design, this study develops three semi-generic criteria based on available kinetic energy, acceleration, and jerk to consider an extensive range of energy harvester designs. The six observed sites are evaluated based on the defined criteria to find optimal implant sites for increasing energy harvesting performance. Finally, an endocardial energy harvester is considered to evaluate the efficiency of the developed semi-generic criteria.

This paper is organized as follows. Section , "Materials and Methods," details the experimental setup, including the motion sensor configuration, in-vivo measurement procedures, post-processing kinematic analysis of recorded heart motion, and the developed energy harvesting criteria. Section , "Results" presents the findings from applying these criteria and evaluates the performance of a conceptual endocardial energy harvester. Finally, Section offers the "Discussions" summarizing the key findings and discussing future research directions.

## Materials and Methods

This section details the experimental setup and methodologies used to characterize heart motion for energy harvesting. This begins with a comprehensive description of the miniaturized 9-degree-of-freedom motion sensor, including its components, custom printed circuit board design, and encapsulation for in-vivo implantation. Following this, the in-vivo animal study procedures are outlined, covering the surgical approach, sensor calibration, and the strategic placement of the sensor at various epicardial sites in a porcine model to capture 3D translational and rotational heart movements across different heart rates. Finally, the section introduces the post-processing kinematic analysis, which transforms the raw sensor data into a fixed reference frame and defines the energy harvesting criteria based on kinetic energy, acceleration, and jerk to evaluate the potential of each implant site.

### Motion Sensor Configuration

The proposed sensor for heart motion measurement must provide accurate and comprehensive motion data. Its configuration includes an accelerometer to measure 3D linear acceleration, a gyroscope to capture the angular velocity of the implant site, and a 3D magnetometer to assess the strength of the local magnetic field. By integrating these measurements through a fusion algorithm, the sensor can accurately determine the 3D orientation of the implant site, ensuring precise motion tracking. The Bosch BNO055 inertial measurement unit (IMU) fulfills these requirements, making it an ideal choice for this application. This compact, advanced sensor is an all-in-one IMU that integrates the accelerometer, gyroscope, and magnetometer into a single device. It also includes a built-in microcontroller to process sensor fusion algorithms, delivering reliable 3D motion and orientation data. Its compact form factor makes it highly suitable for implantation in medical studies, where minimizing size and weight is crucial to prevent interference with natural heart motion. The sensor’s accuracy and bandwidth in fusion mode are detailed in Supplementary Table S1.

**Fig. 1:**
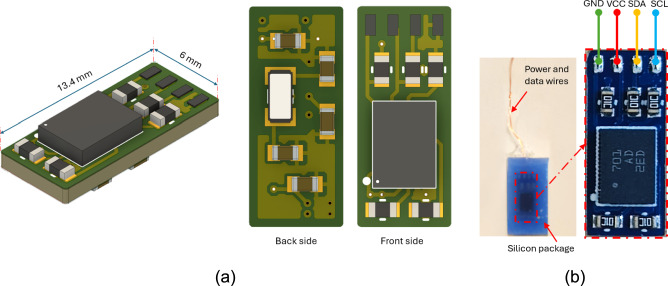
**a** The miniaturized motion sensor with double-sided PCB design, **b** the miniaturized sensor encapsulated by a silicon package (with a blue color), and a zoom-in view of the actual sensor

The BNO055 sensor is integrated with additional electronic components on a printed circuit board (PCB) to function as a complete sensor system in this study. Considering the space-constrained environment of heart motion measurement, a customized double-sided PCB design is utilized to reduce the sensor system's overall dimensions significantly. The miniaturized double-sided PCB’s design with dimensions 13.4 × 6 × 3 mm is shown in Fig. 1(a). This approach allows a more compact and lightweight sensor, minimizing potential tissue impact.

We implanted the miniaturized sensor into the epicardium during the animal test. Fig. 1(b) shows the miniaturized sensor placed into a blue silicone package to prevent possible blood interference and facilitate suturing. The total mass of the motion sensor with its silicone package is 2 g, comparable to commercial ICLPs, and is sufficiently small to avoid impacting heart motion. The miniaturized sensor board requires four primary connections to function: ground (GND), power supply (VCC, typically 3.3V), and two serial communication lines (SCL and SDA) for I2C protocol. These connections are essential for providing power to the sensor and enabling data transfer between the microcontroller and the BNO055 sensor. In this research, the Arduino Due is used as the microcontroller to interface with the BNO055 sensor.

### In-Vivo Measurements

We conducted an in-vivo animal study in a porcine model to measure heart motion. We utilized the porcine model as it is a standard analogue for human cardiac research, primarily due to its high degree of anatomical and physiological similarity to the human heart. We performed open-chest surgery to expose the heart and implant the sensor at different epicardial points and heart rates. We chose the locations for their relevance to typical leadless-pacemaker implantation sites. This study was approved by the Danish Animal Experiments Inspectorate, license no 2021-15-0201-00882. Detailed medical information of the animal test and procedural setup is provided in the Supplementary section S2.

The animal underwent an open-chest surgery to expose the heart and securely implant the motion sensor on different epicardial sites. Before sensor implantation, the motion sensor was carefully calibrated according to the manufacturer’s instructions. Precise data collection was ensured by performing zero-bias correction to eliminate sensor drift, static and dynamic calibration to validate acceleration and angular velocity measurements, and magnetometer calibration to mitigate potential interference from the surgical environment. The operation of the sensor is also sensitive to electrostatic charge; therefore, to eliminate any interference during the animal test, all personnel used an earth wire. All measurements were taken from the same animal, during a single experiment.

**Fig. 2:**
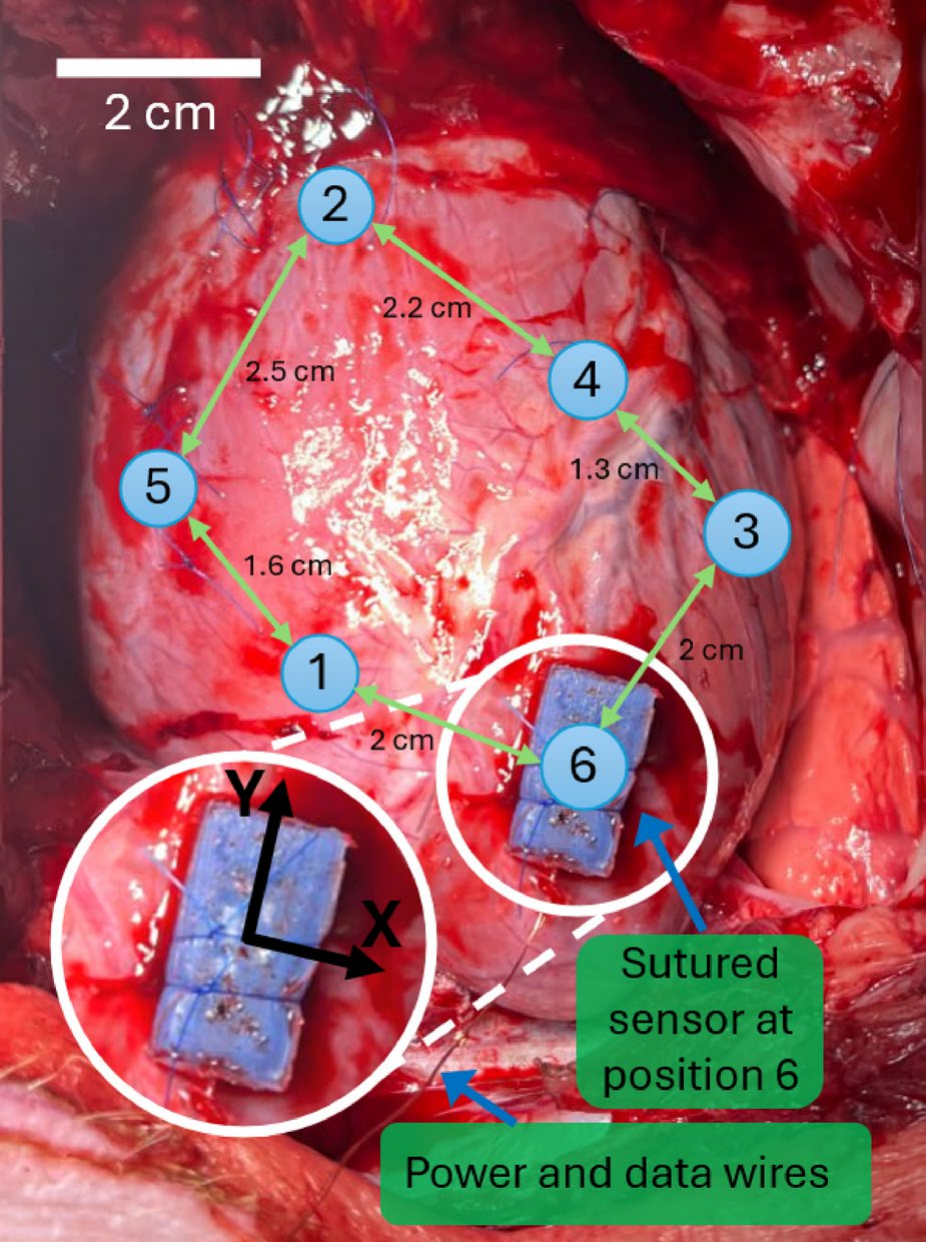
The observed epicardial implant sites (by motion sensor), and the implanted sensor by suturing at position 6, along with the local motion sensor’s coordinate system. The annotated distances reflect relative placement on the 2D view

We sutured the motion sensor (in a biocompatible silicone package) to six specific epicardial locations to measure 3D translational and rotational heart motion. These sites were selected based on their relevance to potential pacemaker implantation. During this animal test, a temporary pacemaker device was introduced through the venous catheter and connected to the heart to control heart rate. This temporary pacemaker allows controlled pacing at different specific rates, ensuring motion data is captured across various physiological conditions. The observed epicardial sites in this investigation are reported in Table 1. Moreover, Fig. 2. illustrates the implant sites over the epicardium with the motion sensor implanted at position 6. The shown relative distances between the implant sites are measured from a 2D projection to document the position at each acquisition, which may differ slightly from the true 3D separations. At implantation, the sensor’s local axes were aligned to cardiac directions: Z is oriented perpendicular to the local epicardial surface (outward); Y was aligned base-to-apex; X is orthogonal to Y and Z, approximating the circumferential/torsional direction.

**Table 1:** The measured motion cases at different epicardial implant sites and heart rates (HR) by sensor

Positions	Placement
1	Mid-Septum right
2	Right ventricle – outflow tract
3	Basal lateral
4	High septum
5	Mid-anterior right ventricle
6	Apex, left ventricle

After implanting the motion sensor, we recorded real-time 3D translational accelerations, rotational velocities, and orientations at 50–70 Hz. We captured high-resolution cardiac motion data across a wide heart rate range (80–130 bpm), with over 20 cardiac cycles acquired per heart rate. Recorded data was transmitted via data cable to an Arduino Due board and subsequently to a computer for post-processing analysis.

### Post-Processing Motion Analysis

**Fig. 3:**
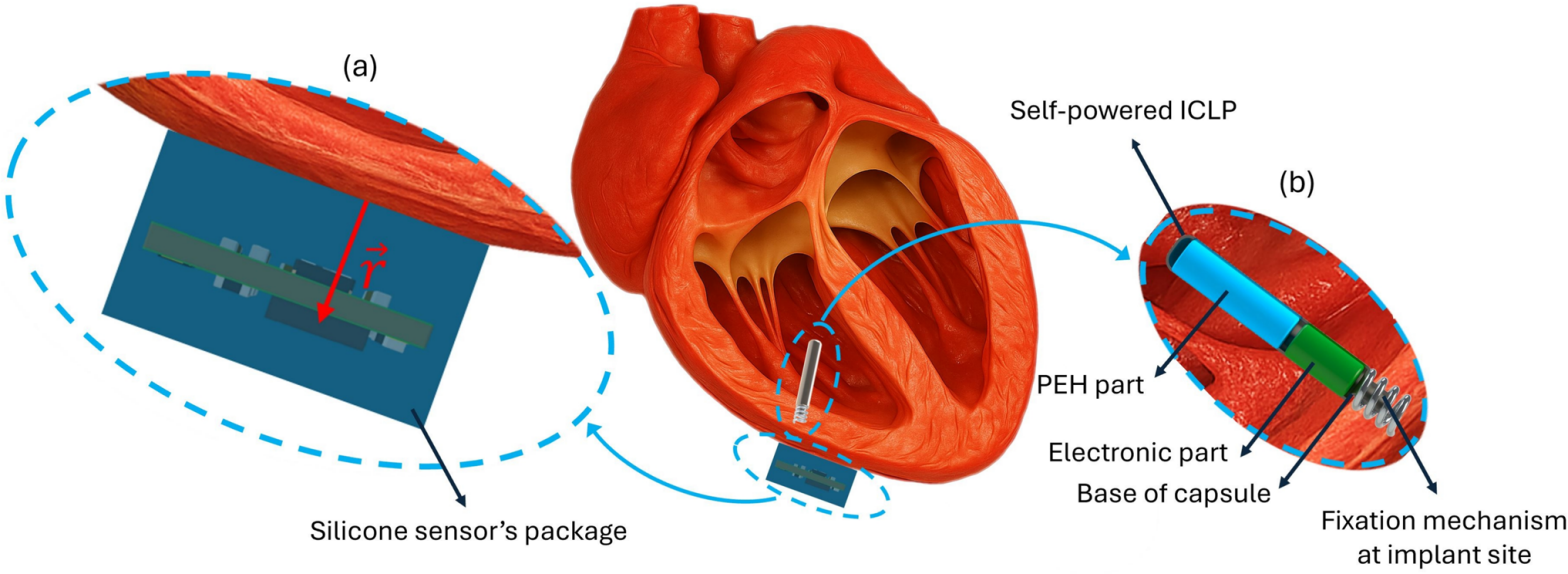
The cross-sectional view of the heart, **a** showing the distance offset vector $\overrightarrow{r}$ between sensor and heart surface, **b** the cross-sectional view of an ICLP with a cylindrical capsule package over the endocardium

The motion sensor measures the 3D linear acceleration and angular velocity in the sensor’s body frame, which follows the orientation of the sensor. It is required to transform measured data from the body frame to the fixed reference frame because the operation of proposed energy harvesters relates to inertial force that should be analyzed in the fixed reference coordinate system. Fig. 3(a) shows the motion sensor implanted over the epicardium with an offset vector $\vec{r}$ from the heart surface. In supplementary section S3, a kinematic analysis is used to derive the corresponding base excitation that would be experienced by a self-powered ICLP through the fixation mechanism shown in Fig. 3(b). As a result, the periodic base excitation, consisting of linear acceleration vector ($\overrightarrow {{a^{\prime}}}_{base} )$ and rotational velocity vector ($\overrightarrow {{\Omega^{\prime}}}$) are computed. To mitigate high-frequency sensor noise, the raw kinematic data were processed using a low-pass filter with a cutoff frequency of 30 Hz. Supplementary Figure S1 shows a sharp decline in signal magnitude beyond 25 Hz, confirming that higher frequencies contain no significant physiological information.

It is supposed that the dimensions of the proposed self-powered ICLP are similar to current commercial pacemaker devices. For instance, the model of Abbott Aveir™ VR is characterized by a length of 38 mm and a diameter of 6.5 mm [2]. Usually, the battery occupies 60-70% of the pacemaker i.e., a cylindrical volume with a length 24.7 mm and diameter 6.5 mm. In the self-powered ICLP concept design, the battery volume can be allocated to the PEH part, as shown in Fig. 3(b). Applying the derived motion vectors ($\overrightarrow {{a^{\prime}}}_{base}$ and $\overrightarrow {{\Omega^{\prime}}}$) to the base of ICLP create a time-dependent and position-dependent distribution of velocity and acceleration over the PEH volume, which should be analyzed for energy harvesting evaluation. The motion of each point within the capsule volume can be calculated either analytically or numerically by methods such as FEM. This research used a time-dependent model in COMSOL Multiphysics for kinematic analysis.

The following analysis aims to identify optimal implant locations across varying heart rates based on energy harvesting potential. The optimal implant site(s) may vary depending on the specific energy harvester’s design because the energy harvesting potential of each implant site depends on the energy harvester's properties. It is thus desirable to establish energy harvesting criteria that facilitate examining each implant site’s potential based on a broad range of energy harvester configurations.

### Energy Harvesting Criteria

In this study, several criteria are defined to encompass essential energy harvesting factors for a wide range of energy harvester designs. Specifically, three groups of criteria are developed based on (I) available kinetic energy (velocity), (II) acceleration, and (III) jerk, or acceleration derivative. All measured cases are evaluated against these criteria to predict each location’s energy harvesting potential at various heart rates. Finally, a specific energy harvester design is considered to examine the validity of the defined criteria.

### Criteria group I: The Available Kinetic Energy

PEHs convert the kinetic energy of motion to electrical energy, so the total kinetic energy within the available capsule volume, $\mathbb{V}$, over a cycle of motion can be considered an energy harvesting criterion. As the inertial mass of the energy harvester is independent of the motion, only the magnitude of velocity at each point in the capsule volume contributes to a criterion for the evaluation of motion. Therefore, the first criteria group considers the time-averaged and volume-averaged velocity component magnitudes within $\mathbb{V}$ during one motion cycle, which correlates to the kinetic energy available for energy harvesting. This criteria group is expressed based on velocity components $v_{x} , v_{y} , v_{z}$ as:

1 $$Cr_{Ix} = \frac{1}{T}\mathop \smallint \limits_{0}^{T} \frac{1}{{ \mathbb{V}}}\iiint\limits_{{ \mathbb{V}}} {\left| {v_{x} \left( {x,y,z,t} \right)} \right|} d{ \mathbb{V}}dt ,\left[ {m/s} \right]$$

with corresponding definitions for $Cr_{Iy}$ and $Cr_{Iz}$. Then, using the velocity magnitude:

2 $$Cr_{I} = \frac{1}{T}\mathop \smallint \limits_{0}^{T} \frac{1}{{ \mathbb{V}}}\iiint\limits_{{ \mathbb{V}}} {\sqrt {v_{x}^{2} + v_{y}^{2} + v_{z}^{2} } } \, d{ \mathbb{V}}dt ,\left[ {m/s} \right]$$

where $T$ represents the duration of a cardiac cycle.

### Criteria Group II: The Available Acceleration

In addition to the available kinetic energy (considered in the first criteria group), the available acceleration, and hence inertial force, is important in energy harvesting. Therefore, a second criteria group focuses on acceleration available within the PEH volume, which represents inertial loading potential. The time- and volume-averaged acceleration for *x*-component over a cycle of motion are given by:

3 $$Cr_{IIx} = \frac{1}{T}\mathop \smallint \limits_{0}^{T} \frac{1}{{ \mathbb{V}}}\iiint\limits_{{ \mathbb{V}}} {\left| {a_{x} \left( {x,y,z,t} \right)} \right| }d{ \mathbb{V}}dt \,,\,\,\left[ {m/s^{2} } \right]$$

with corresponding definitions for $Cr_{IIy}$ and $Cr_{IIz}$. Then, the overall acceleration magnitude:

4 $$Cr_{II} = \frac{1}{T}\mathop \smallint \limits_{0}^{T} \frac{1}{{ \mathbb{V}}}\iiint\limits_{{ \mathbb{V}}} {\sqrt {a_{x}^{2} + a_{y}^{2} + a_{z}^{2} } } \, d{ \mathbb{V}}dt ,\left[ {m/s^{2} } \right]$$

### Criteria GROUP III: The Available Jerk

PEHs convert an applied mechanical strain or stress to electrical output. In inertial systems, this is dependent on base acceleration. However, constant acceleration leads to a constant piezoelectric charge in such systems, whereas charge flow is required for the extraction of electrical energy. Thus, in addition to the level of base acceleration, the time-varying acceleration is crucial for energy harvesting since constant stress or strain in the piezoelectric element does not lead to electrical power output [22, 23]. In this way, the time derivative of acceleration, known as “jerk”, can be considered an effective factor in energy harvesting analysis, reflecting the dynamic variation necessary to induce charge flow in piezoelectric elements. Thus, the third criteria group ($Cr_{IIIx} , Cr_{IIIy} , Cr_{IIIz} , Cr_{III}$) examines the time-averaged and volume averaged available jerk within the PEH volume during one cardiac cycle as follows:

5 $$Cr_{IIIx} = \frac{1}{T}\mathop \smallint \limits_{0}^{T} \frac{1}{{ \mathbb{V}}}\iiint\limits_{{ \mathbb{V}}} {\left| {j_{x} \left( {x,y,z,t} \right)} \right| }d{ \mathbb{V}}dt ,\left[ {m/s^{3} } \right]$$

where $j_{x} = \frac{d}{dt}a_{x}$, with corresponding definitions for $Cr_{IIIy}$ and $Cr_{IIIz}$. Then, considering the overall jerk magnitude:

6 $$Cr_{III} = \frac{1}{T}\mathop \smallint \limits_{0}^{T} \frac{1}{{ \mathbb{V}}}\iiint\limits_{{ \mathbb{V}}} {\sqrt {j_{x}^{2} + j_{y}^{2} + j_{z}^{2} } } \, d{ \mathbb{V}}dt ,\left[ {m/s^{3} } \right]$$

In the defined criteria groups, the effect of both translational and rotational motions was considered. To estimate the contribution level of the 3D cardiac rotational motion in the criteria values, an expression $f_{{\Omega }}$ is defined to compute the fraction of each criterion’s value arising due to rotational motion. Thus, for any of the assessment criteria $Cr_{*}$ the corresponding value of the same criterion evaluated without considering rotational motion $Cr_{{* - {\Omega }}}$ is computed and $f_{{\Omega }}$ is calculated by:

7 $${\mathrm{f}}_{{\Omega }} = \frac{{\left| {{\mathrm{Cr}}_{*} - {\mathrm{Cr}}_{{{*} - {\Omega }}} } \right|}}{{{\mathrm{Cr}}_{*} }} \times 100{\text{ \% }}$$

### Endocardial Energy Harvesting

This section studies an endocardial energy harvester under heart motion at the measured sites. Moreover, the effectiveness of the defined energy harvesting criteria is evaluated in this case study. The proposed energy harvester’s design is based on a conventional cantilever piezoelectric beam configuration, as shown in Fig. 6. This design features a spatial distribution of PEH beams that enables the energy harvester to benefit from motion patterns throughout the cylinder volume. There are 25 piezoelectric beams with specific label numbers arranged in the proposed cylindrical space for the PEH part. Each piezoelectric beam consists of a bottom substrate layer (silicon), an upper piezoelectric layer (PZT-5H), and a tip mass (0.2 mg). The thickness of each piezoelectric and substrate layer is 20 µm. Each piezoelectric beam is connected to a separate electrical resistance ($$R_{L} )$$ to measure the maximum energy extracted by the individual piezoelectric beams. The distributed piezoelectric beams in this design facilitate the investigation of energy harvesting levels at various locations within the energy harvester's volume.

In the literature [22, 23], an analytical model has been developed to predict the response of cantilever piezoelectric energy harvesters (with bending motion) subjected to transverse base excitation (in the *z*-direction). In this study, the *z*-direction is aligned with the normal axis of the heart surface. The voltage generated under a harmonic transverse excitation with an amplitude $W_{0}$ and frequency $\omega$ ($w_{b} \left( t \right) = W_{0} e^{j\omega t}$) is given by:

8 $$V\left( t \right)\; = \,\frac{{\mathop \sum \nolimits_{i\,\, = \,1}^{\infty } \frac{{ - j\omega F_{i} \theta_{i} }}{{ - \omega^{2} + 2j\xi_{i} \omega_{i} \omega + \omega_{i}^{2} }}}}{{j\omega C_{p} + \frac{1}{{R_{L} }} + \mathop \sum \nolimits_{i = 1}^{\infty } \frac{{j\omega \theta_{i}^{2} }}{{ - \omega^{2} + 2j\xi_{i} \omega_{i} \omega + \omega_{i}^{2} }}}}e^{j\omega t} ,\left[ V \right]$$

where parameters $\theta_{i}$ and $\xi_{i}$ represent the equivalent modal electromechanical coupling and damping ratio, respectively. Moreover, the parameters $\omega_{i}$, $C_{p}$, and $R_{{\mathrm{L}}}$ are the ith undamped natural frequency, piezoelectric capacitance, and the connected electrical load’s resistance, respectively. The $F_{i}$ represent the equivalent modal force of the proposed piezoelectric beam with length $L$, width $b$, and tip mass $M_{t}$, which can be expressed as

9 $${\mathrm{F}}_{{\mathrm{i}}} = - \left[ {\left( {{\uprho }_{{\mathrm{b}}} {\mathrm{h}}_{{\mathrm{b}}} + 2{\uprho }_{{\mathrm{p}}} {\mathrm{h}}_{{\mathrm{p}}} } \right)\mathop \smallint \limits_{0}^{{\mathrm{L}}} {\mathrm{b}}\left( {\mathrm{x}} \right)\phi_{{\mathrm{i}}} \left( {\mathrm{x}} \right){\mathrm{dx}} + {\mathrm{M}}_{{\mathrm{t}}} \phi_{{\mathrm{i}}} \left( {\mathrm{L}} \right)} \right]{\mathrm{W}}_{0} {\upomega }^{2} = - {\mathrm{mW}}_{0} {\upomega }^{2}$$

The constants $\rho_{b}$, $\rho_{p}$, $h_{b}$, and $h_{p}$ denote the density and thickness of the substrate and piezoelectric layers, respectively. The function $\phi_{i} \left( x \right)$. is ith mode shape. Consequently, the fundamental frequency of the proposed piezoelectric beams is significantly higher than the harmonics present in heart motion ($\omega_{1} \gg \omega )$. due to their low length. In this case, considering only the fundamental mode is sufficient; the higher-order resonant modes can be neglected. Thus, Eq. (8) simplifies to:

10 $$V(t)\, = \,\frac{{ - jm\theta_{1} }}{{\omega_{1}^{2} (j\omega C_{p} + \frac{1}{{R_{L} }} + \frac{{j\omega \theta_{1}^{2} }}{{\omega_{1}^{2} }})}}j_{z} (t)$$

where the variable $j_{z} \left( t \right)$. is the applied transverse jerk given by

11 $$j_{z} \left( t \right) = - W_{0} \omega^{3} e^{j\omega t} ,\left[ {m/s^{3} } \right]$$

The voltage output $V\left( t \right)$ is proportional to the transverse jerk $j_{z} \left( t \right)$ for each individual harmonic of the motion. Moreover, it can be shown that the effect of other jerk components $j_{x} \left( t \right)$ and $j_{y} \left( t \right)$ is negligible for a piezoelectric cantilever beam lying in the x-y plane. Large rotations between the body frame and fed frame can produce significant jerk components $j_{x} \left( t \right)$ and $j_{y} \left( t \right)$. However, due to the limited rotation of the heart surface, the effects of $j_{x} \left( t \right)$ and $j_{y} \left( t \right)$ are expected to be negligible for the proposed energy harvester in this study.

The instantaneous generated power of the *i*th piezoelectric beam under a specific heart motion excitation is

12 $$P_{i } \left( t \right) = \frac{{V_{i}^{2} \left( t \right)}}{{R_{L} }} ,\left[ W \right]$$

where $V_{i} \left( t \right)$ is the voltage of the *i*th piezoelectric beam. The overall harvested power under the considered heart motion excitation is computed by summing over all beams:

13 $$p_{total} \left( t \right) = \mathop \sum \limits_{i = 1}^{25} P_{i} \left( t \right) ,\left[ W \right]$$

Normalizing with respect to the peak instantaneous power gives:

14 $$P_{total} \left( t \right) = \frac{{p_{total} \left( t \right)}}{{\max \left( {p_{total} } \right)}}$$

**Fig. 4:**
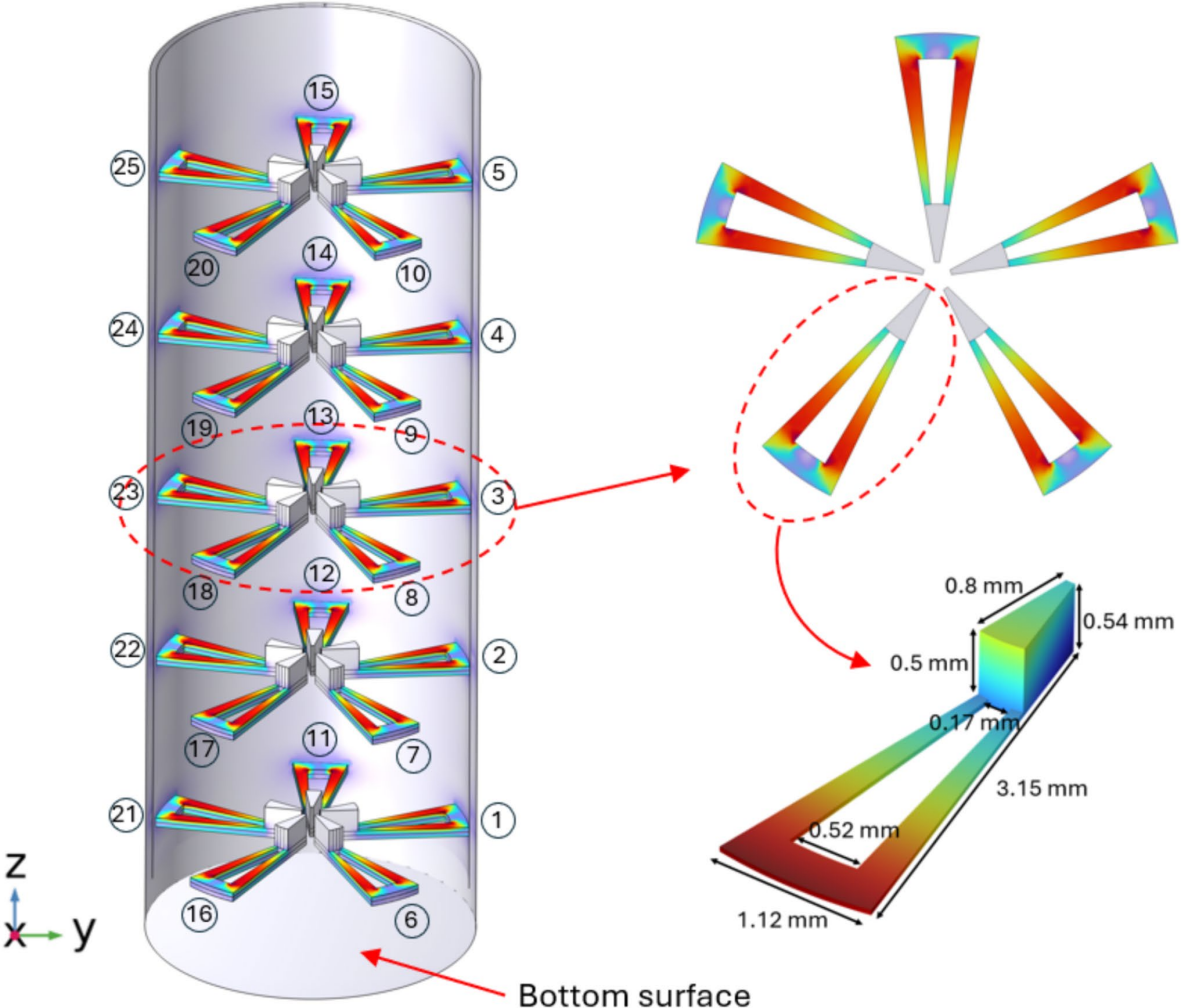
The proposed endocardial energy harvester design, consisting of 25 individual piezoelectric beams, subjected to heart motion applied through the bottom surface; the color contour maps the von Mises stress distribution

A finite element model of the energy harvester (illustrated in Fig. 4) is used to examine electrical output with measured heart motions as input. It is of interest to compare the normalized instantaneous power $P_{total,m} \left( t \right)$ to the time dependent volume averages of velocity, acceleration, and jerk given by:

15 $$\overline{{v_{x} }} \left( t \right) = \frac{1}{v}\iiint\limits_{v} {\left| {v_{x} \left( {x,y,z,t} \right)} \right| }dv ,\left[ {m/s} \right]$$

along with corresponding expressions for the *y* and *z* directions, for accelerations ($\overline{{a_{x} }} \left( t \right)$ etc.) and for jerk ($\overline{{j_{x} }} \left( t \right)$ etc.).

This finite element model is conducted in COMSOL Multiphysics using the solid mechanics, electrostatics, and electrical circuits modules. Each piezoelectric beam has an electrical connection to a separate resistance $R_{L}$ = 50 kΩ through individual voltage terminals. The piezoelectric beams are connected to the cylinder through a fixed constraint. Thin electrically conductive layers are applied to both the upper and bottom surfaces of the piezoelectric layers to collect and transport electrical charges generated within the material subjected to mechanical stress. The isotropic material properties of density, Young’s modulus, and Poisson ratio for the silicon layer are 2329 kg/m^3^, 170 GPa, and 0.28, respectively. Moreover, the material properties of the piezoelectric layer (PZT-5H) are presented in the Appendix. The isotropic loss factor $\eta_{s} = 0.01$ was considered as a mechanical damping.

## Results

This section presents a comprehensive analysis of the energy harvesting potential at various epicardial implant sites and heart rates, using the criteria defined in the preceding sections. The evaluation is based on three groups of criteria: available kinetic energy (velocity), acceleration, and jerk. The results from these criteria are discussed, highlighting how different implant sites perform under varying physiological conditions. Additionally, the influence of rotational cardiac motion on the criteria values is assessed. Finally, an endocardial energy harvester design is analyzed to validate the effectiveness of the established criteria in predicting power output from heart motion.

### Analysis of energy harvesting potential of implant sites

The heart motion across all implant sites (as stated in Table 1) is comprehensively analyzed using the defined energy harvesting criteria at different heart rates to provide a statistical assessment of measurement repeatability and uncertainty. The 20 cardiac cycles recorded for each case were grouped into heart-rate bins for each of the six implant positions. The detailed results are provided in Supplementary Section S4.

**Fig. 5:**
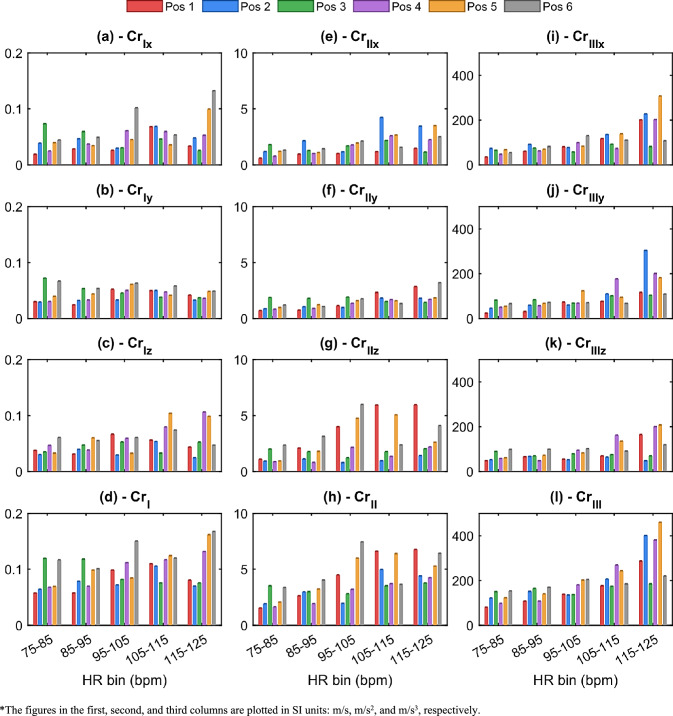
The median values of criteria at different implant sites and heart-rate bins; kinetic energy criteria: **a** $C{r}_{Ix}$, **b** $C{r}_{Iy}$, **c** $C{r}_{Iz}$, **d** $C{r}_{I}$, acceleration criteria: **e** $C{r}_{IIx}$, (f) $C{r}_{IIy}$, **g** $C{r}_{IIz}$, **h** $C{r}_{II}$, and jerk criteria: **i** $C{r}_{IIIx}$, **j** $C{r}_{IIIy}$, **k** $C{r}_{IIIz}$, **I** $C{r}_{III}$. Position 6 (grey) and Position 5 (orange) generally show the highest median values across all criteria. In contrast, Position 2 (blue) consistently shows the lowest motion levels

To compare the energy-harvesting potential of implant sites, Fig. 5 summarizes the median values of the energy-harvesting criteria for each heart rate bin. The first, second, and third columns in this figure show the results for the first, second, and third groups of criteria, respectively. The most consistent trend across all 12 graphs is a positive correlation between complexity and heart rate. However, some criteria are not considerably affected by heart rate, such as $Cr_{Iy}$.

Due to the high complexity and variations in Fig. 5, it is complicated to provide a comprehensive analysis or consistent patterns across all directions and implant sites. In this way, Table 2 summarizes the directional mean criteria values ($\overline{Cr}$) in all 12 subplots in Fig. 5 throughout all implant sites and heart-rate bins. As shown in the supplementary material and Fig. 2, the x-, y-, and z-axes of the sensor’s coordinate system were aligned with the heart’s torsional, longitudinal, and normal motions throughout all measurements. Table 2 indicates that the first and third criteria are maximal in the torsional direction, whereas the second criterion is maximal in the normal direction. Moreover, the averaged results show that the longitudinal direction has the lowest motion level; however, the third criteria group’s result for implant site 2 (in Fig. 5) is maximal in the longitudinal direction.

**Table 2:** Directional average of the criteria values ($\overline{Cr }$) in Fig. 1 throughout all implant sites and heart-rate bins

Criteria group	Criteria values in different directions			
	Torsional (x)	Longitudinal (y)	Normal (z)	Total
First (m/s)	$\overline{C{r }_{Ix}}$= 0.051	$\overline{C{r }_{Iy}}$= 0.044	$\overline{C{r }_{Iz}}$= 0.048	$\overline{C{r }_{I}}$= 0.10
Second (m/s^2^)	$\overline{C{r }_{IIx}}$= 1.830	$\overline{C{r }_{IIy}}$= 1.56	$\overline{C{r }_{IIz}}$= 2.46	$\overline{C{r }_{II}}$ 3.94
Third (m/s^3^)	$\overline{C{r }_{IIIx}}$=101.7	$\overline{C{r }_{IIIy}}$= 93.6	$\overline{C{r }_{IIIz}}$= 89.9	$\overline{C{r }_{III}}$= 192.9

To facilitate a clearer, normalized comparison of the sites, the median criteria values presented in Figure 5 were converted into a scoring system for Table 3. For each three multidirectional criteria ($\overline{{Cr_{I} }}$, $\overline{{Cr_{II} }}$, $\overline{{Cr_{III} }}$) and each specific heart-rate bin, a position's score was calculated as the ratio of its median value to the sum of the median values from all six positions, then multiplied by 100. This score effectively represents each site's percentage contribution to the total motion captured across all sites for that specific criterion and heart rate. The highlighted cells within the table identify the optimal implant site for each criterion within each specific heart-rate (HR) range. This data illustrates that the best-performing location is not consistent, but rather changes depending on both the heart rate and the specific motion characteristic (velocity, acceleration, or jerk) being measured. For example, while Position 6 (Apex, left ventricle) and Position 5 (Mid-anterior right ventricle) frequently show high scores, Position 3 (Basal lateral) is optimal for acceleration and jerk criteria at lower heart rates, and Position 4 (High septum) is optimal for the jerk criterion in the 105-115 bpm range. For a detailed breakdown of the real kinematic values used to derive these scores across different heart rate ranges, please refer to the Supplementary Table S2.

**Table 3:** The average scores of the criteria of $\overline{C{r }_{I}}$, $\overline{C{r }_{II}}$, and $\overline{C{r }_{III}}$ at each heart-rate bin and each implant site

Implant sites	Implant sites’ performance score (0-100) by at different heart-rate bins (BPM)														
	75–85			85–95			95–105			105–115			115–125		
	$\overline{{Cr }_{I}}$	$\overline{{Cr }_{II}}$	$\overline{{Cr }_{III}}$	$\overline{{Cr }_{I}}$	$\overline{{Cr }_{II}}$	$\overline{{Cr }_{III}}$	$\overline{{Cr }_{I}}$	$\overline{{Cr }_{II}}$	$\overline{{Cr }_{III}}$	$\overline{{Cr }_{I}}$	$\overline{{Cr }_{II}}$	$\overline{{Cr }_{III}}$	$\overline{{Cr }_{I}}$	$\overline{{Cr }_{II}}$	$\overline{{Cr }_{III}}$
Pos 1	11.6	10.9	11.0	11.0	14.8	12.8	16.5	17.3	13.9	17.3	24.2	14.5	11.1	21.5	16.5
Pos 2	13.0	13.6	16.8	15.0	16.7	18.0	12.0	7.5	13.5	14.0	12.6	14.0	14.6	15.8	11.9
Pos 3	24.1	25.1	20.7	22.6	16.8	19.6	13.7	10.8	13.7	11.9	12.9	14.3	10.5	12.0	10.6
Pos 4	13.7	11.6	13.4	13.3	10.8	12.8	18.7	12.4	18.1	18.4	13.6	22.1	18.2	13.5	21.9
Pos 5	14.0	14.7	16.9	18.8	18.2	16.7	14.1	23.1	20.2	19.6	23.4	20.0	22.4	16.8	26.5
Pos 6	23.6	24.1	21.2	19.2	22.7	20.2	25.1	28.8	20.6	18.9	13.3	15.2	23.2	20.4	12.6

**Contribution of Rotational Motion**

**Fig. 6:**
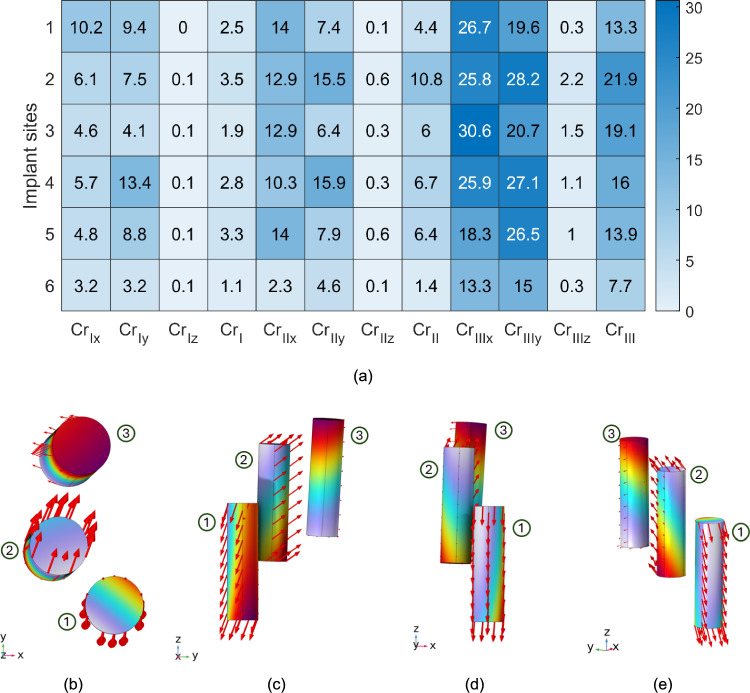
**a** The effect of rotational motion (${f}_{\Omega })$ in each criterion at different implant sites, the distribution of jerk magnitude within the energy harvester’s cylindrical volume along with velocity arrow lines at different time instants ((1) t = 3 ms, (2) t = 90 ms, and (3) t = 200 ms) in **b** x-y, **c** y-z,**d** z-x, and **e** perspective views

To assess the contribution level of the 3D rotational motion in energy harvesting criteria, the average of $f_{{\Omega }}$ values (defined in Eq. (7) of some measurements with different heart rates at individual implant sites for each criterion are reported in Fig. 6(a). The results indicate that although the velocity, acceleration, and jerk components in the *x*- and *y*-directions are significantly affected by rotation motion, the components in the *z*-direction are only slightly changed by rotation. This arises because the rotation angles are relatively small (only a few degrees), and the offset between the epicardium and the sensor remains approximately in the *z*-direction throughout the cardiac cycle. Therefore, the criteria $Cr_{Iz}$, $Cr_{IIz}$, and $Cr_{IIIz}$ are not greatly influenced by rotational motion (as shown in Fig. 6(a)).

The motion of the proposed energy harvester’s volume (at different time instants) is analyzed subject to a measured cardiac motion. The resulting distribution of jerk magnitude over this volume is presented in Fig. 6(b–e), along with velocity arrow lines under base excitation ($\overrightarrow {{a^{\prime}}}_{base} \left( t \right)$ and $\overrightarrow {{\Omega^{\prime}}} \left( t \right)$) with different views at time instants: (1) *t* = 3.2 ms, (2) *t* = 90 ms, and (3) *t* = 200 ms. The shown variation in jerk distribution illustrates the effect of rotational motion on the energy harvester’s kinematics. The effect of rotational motion in instantaneous power generation is shown in Supplemental Figure S9.

### Performance of Energy Harvesting Criteria

**Fig. 7:**
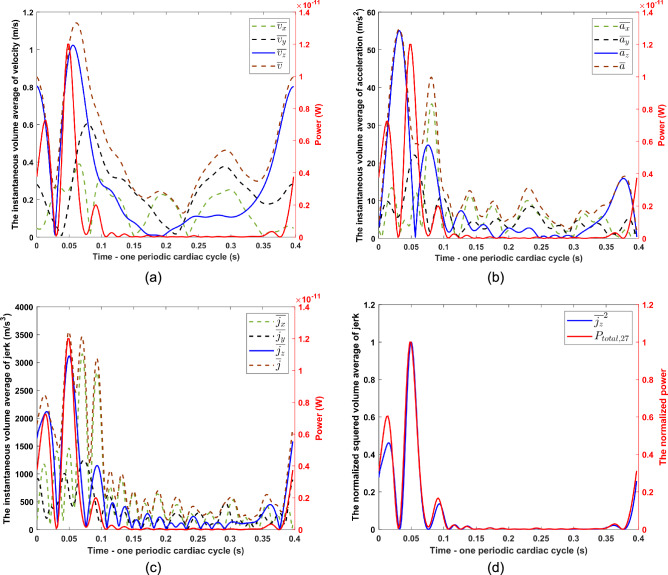
The comparison of normalized whole power against the **a** instantaneous volumetric absolute of velocity, **b** acceleration, **c** jerk, and **d** normalized squared transverse jerk ${\overline{{j }_{z}}}^{2}(t)$. A direct comparison shows that the normalized instantaneous power correlates very closely with the normalized squared transverse jerk ($\overline{{j }_{z}^{2}}$), validating ${Cr}_{IIIz}$ as a predictive criterion for this harvester design

This section evaluates the performance of the defined criteria in predicting the energy-harvesting potential of implant sites. In this way, the output of the energy harvester shown in Fig. 4 is examined under a specific measured heart motion to determine which motion measure best correlates with instantaneous power generation. The results of the instantaneous volume average of velocity, acceleration, and jerk defined in Eq. (14) and corresponding expressions are presented in Fig. 7(a–c), respectively. Fig. 7(a),(b) indicate that there is no clear correlation between instantaneous power and either velocity or acceleration. However, Fig. 7(c) shows that the variation of instantaneous power aligns with the measure of available jerk*.* Particularly, the peaks of instantaneous power occur when $\overline{{j_{z} }} \left( t \right)$ reaches its peaks. However, there is no explicit correlation between instantaneous power and in-plane jerk components $\overline{{j_{x} }} \left( t \right)$ and $\overline{{j_{y} }} \left( t \right)$.

This directional sensitivity is a direct consequence of the energy harvester design employed in this study. While cardiac motion is inherently three-dimensional, the planar cantilever beams fig. 4 act as directional filters. Due to the high aspect ratio of the piezoelectric beams, their primary mode of voltage generation is bending induced by inertial forces applied perpendicular to the beam surface. Consequently, the normal jerk component ($\overline{{j_{z} }} \left( t \right)$) acts as the dominant driving force, inducing significant deflection and strain, whereas the tangential ($\overline{{j_{x} }} \left( t \right)$) and shear ($\overline{{j_{y} }} \left( t \right)$) components act along the stiffer axes of the beam, contributing negligibly to power output.

Eq. (10) and Eq. (11) suggest that the total generated power is proportional to the square of the instantaneous volume-averaged transverse jerk magnitude,

16 $$P_{total} \left( t \right) \propto \overline{{j_{z} }}^{2} \left( t \right)$$

To evaluate this correlation, the squared transverse jerk normalized to its peak value is compared with the normalized power $\overline{P}_{total} \left( t \right)$ in Fig. 7(d). The result demonstrates that the energy harvester’s instantaneous power correlates very closely with the instantaneous $\overline{{j_{z} }}^{2} \left( t \right)$. Thus, it can be expected that the energy harvesting criterion $Cr_{IIIz}$ is of value in predicting the energy harvesting potential of different locations.

**Fig. 8:**
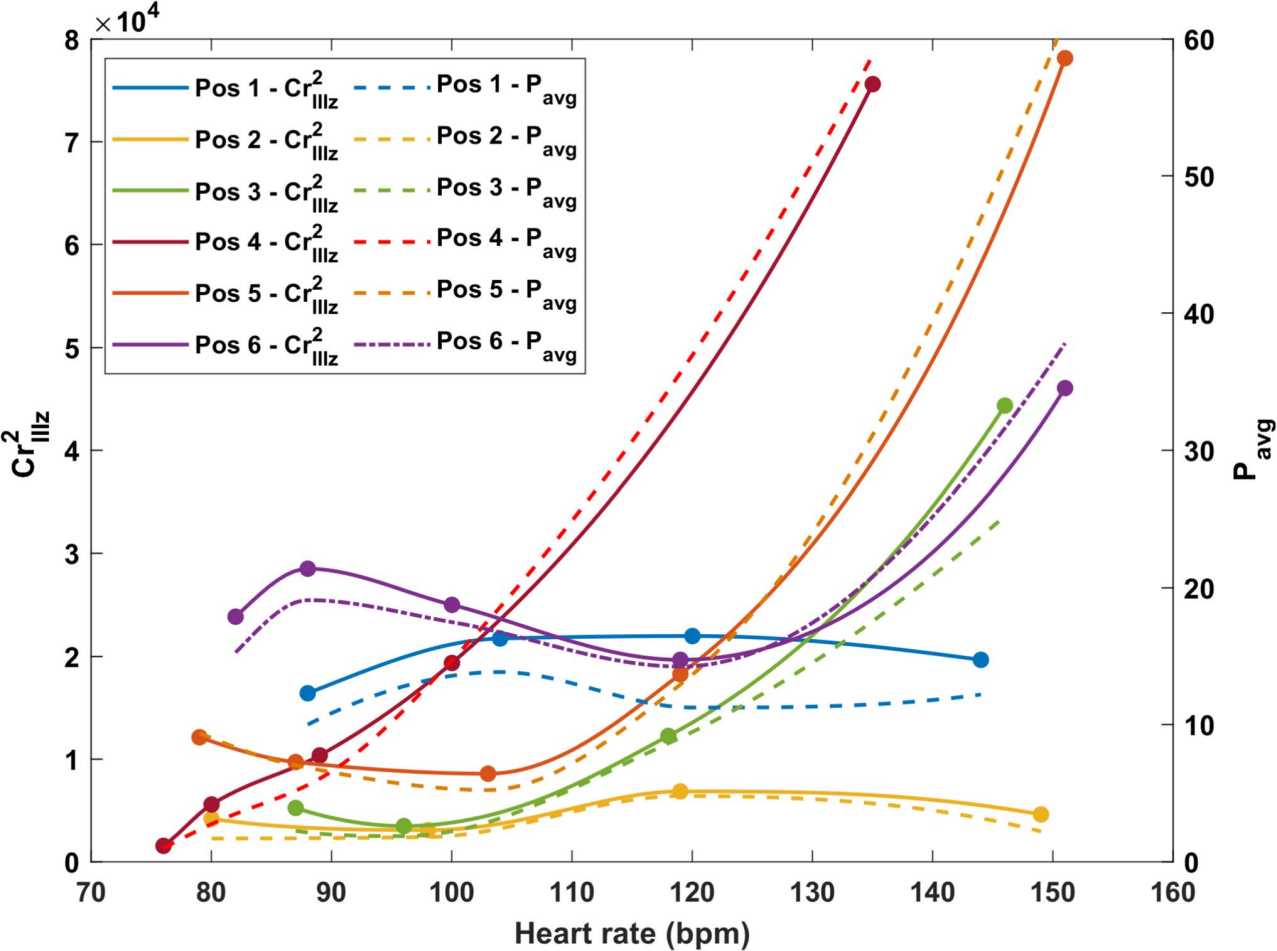
The normalized average power ${P}_{avg}$ compared to $C{r}_{IIIz}^{2}$ at different sites and heart rates, confirming that ${Cr}_{IIIz}$ is a valid and efficient proxy for site selection for this harvester type

Some heart motion measurements at different implant sites and heart rates are considered, and the corresponding average power (during the cardiac cycle) is computed. The derived values are normalized to the lowest average power value, and the normalized values ($P_{avg}$) are shown in Fig. 8 and compared against the corresponding $Cr_{IIIz}^{2}$. The results indicate that there is a close correlation between the normalized average power $P_{avg}$ and the predicted values by $Cr_{IIIz}^{2}$. Thus, the expression in Eq. (15) can be extended to $P_{avg} \propto Cr_{IIIz}^{2}$ for this specific energy harvesting configuration.

It should be noted that the average power values in Fig. 8 were normalized to a baseline of 0.08 fW, reflecting the unoptimized nature of the validation model (a planar cantilever without proof mass). While these absolute levels are insufficient for powering functional intracardiac devices, they successfully serve their intended purpose as a comparative index. To transition from this theoretical validation to a practical clinical application, the energy harvester architecture must be significantly optimized, specifically by adjusting the substrate and piezoelectric layer dimensions, incorporating optimal proof mass to tune the resonant frequency, and optimizing the electrical load resistance to match the source impedance.

## Discussion

This result facilitates evaluating the relative energy harvesting potential of implant sites without requiring an electromechanical analysis of the energy harvester. It demonstrates that, for certain designs of inertial energy harvester, the energy harvesting potential of implant sites on the heart can be assessed by analyzing the motion to find the total available jerk rather than performing an electromechanical analysis of the energy harvester itself at individual implant sites and heart rates. Indeed, the defined criteria provide an efficient analytical framework for evaluating each implant site, significantly decreasing computational load and time.

It is important to note that the dominance of the transverse jerk ($\overline{{j_{z} }}$) observed in this study is specific to the planar cantilever geometry used for validation. While the tangential and shear jerk components ($\overline{{j_{x} }} , \overline{{j_{y} }}$) were excluded from the current power analysis due to the directional sensitivity of cantilever beams, these kinematic components remain significant in the overall cardiac motion profile. Future harvesting designs utilizing 3D structures, spiral beams, or multi-axis coupled oscillators could effectively capture these in-plane motions. By exploiting the total jerk vector ($j_{total}$) rather than just the transverse component, such advanced architectures could potentially increase the total power density and harvest energy from a wider range of epicardial locations.

Although the third criteria group accurately predicts the behavior of certain inertial energy harvester designs, wherein jerk is the dominant source of current flow, it should be emphasized that the appropriate criterion depends on the operating principle of the specific energy harvester. For instance, the available kinetic energy (determined by the first criteria group) may be the most effective criterion for endocardial energy harvester designs based on impact, as investigated in previous work [12]. In this way, Table 4 presents the average of both real kinematic values and scores in Table 3 at each implant site. These average values are used to establish a ranking of the implant sites based on energy harvesting potential estimated by each multidirectional criterion ($Cr_{I}$, $Cr_{II}$, and $Cr_{III}$) over the heart rate range 75-125 bpm.

**Table 4:** The scores and ranking of the observed positions based on energy harvesting capability evaluated by multidirectional criteria

Implant sites	Average real values			Average score			Overall score	Ranking
	${Cr}_{I}$(m/s)	${Cr}_{II}$(m/s^2^)	${Cr}_{III}$(m/s^3^)	${Cr}_{I}$	${Cr}_{II}$	${Cr}_{III}$		
Pos 1	0.081	4.398	157.2	13.5	17.7	13.7	45.0	**5**
Pos 2	0.082	3.045	156.3	13.7	13.2	14.8	41.8	**6**
Pos 3	0.094	3.315	161.4	16.6	15.5	15.8	47.9	**3**
Pos 4	0.099	2.939	206.4	16.5	12.4	17.7	46.5	**4**
Pos 5	0.107	4.584	232.7	17.8	19.2	20.1	57.1	**2**
Pos 6	0.131	4.977	185.9	22.0	21.9	18.0	61.8	**1**

^*^Pos 1: Mid-Septum right, Pos 2: Right ventricle – outflow tract, Pos 3: Basal lateral, Pos 4: High septum, Pos 5: Mid-anterior right ventricle, Pos 6: Apex, left ventricle.

Based on the analysis in Table 4, which provides the average scores for all criteria across the entire 75-125 bpm heart rate range, a clear ranking of the implant sites by energy harvesting potential can be established. The results from the different criteria groups are largely consistent in identifying the most and least suitable locations. Position 6 (Apex, left ventricle) achieves the highest overall score (61.8), ranking it as the most promising site for an energy harvesting implant. It is followed by Position 5 (Mid-anterior right ventricle), which ranked second with a score of 57.1. Conversely, Position 2 (Right ventricle - outflow tract) received the lowest overall score (41.8), indicating it is the least suitable location among those tested. The complete ranking, in descending order of suitability based on the overall score, is: Position 6, Position 5, Position 3 (Basal lateral), Position 4 (High septum), Position 1 (Mid-Septum right), and Position 2.

Moreover, to validate the rankings derived from this scoring system, a rigorous statistical analysis was performed using the Kruskal-Wallis test followed by Bonferroni post hoc comparisons. The statistical results (presented in Supplementary Section S6) confirm that the sites identified here as 'Rank 1' (e.g., Position 6 for velocity/acceleration) exhibit statistically significant superiority (p < 0.05) over lower-ranked positions. This confirms that the scoring system provides a reliable, accessible metric for site selection.

Beyond energy optimization, the clinical translation of this technology requires strict adherence to biocompatibility and durability standards. Since the piezoelectric material used in this model (PZT-5H) contains lead, direct contact with biological tissue poses a toxicity risk. Consequently, the final device design must incorporate robust hermetic encapsulation using materials such as medical-grade titanium to isolate the active element. Moreover, the mechanical durability of the harvester is critical; with the average heart beating approximately 40 million times per year, the device must survive over 400 million cycles to achieve a 10-year lifespan. Future structural optimization must therefore prioritize keeping the dynamic strain levels well below the fatigue endurance limits of both the piezoelectric layer and the substrate.

In conclusion, this study presented a novel approach for characterizing in-vivo cardiac motion to optimize kinetic-based energy harvesting for intracardiac implants. A 9-DOF motion sensor was miniaturized and implanted over the epicardium in an animal model to comprehensively measure and analyze 3D translational and rotational heart motion across multiple epicardial implant sites and heart rates. Three criteria groups based on available kinetic energy (velocity), acceleration, and jerk, were developed to examine the energy harvesting performance of each implant site at different heart rate ranges. Moreover, a theoretical model of an endocardial energy harvester based on distributed cantilever piezoelectric beams was proposed in order to evaluate the energy harvesting output at various implant sites and heart rates. The results highlight the importance of site selection for maximizing energy harvesting potential, with the left ventricular apex emerging as a promising location for jerk-based energy harvesters. The proposed jerk-based criterion effectively predicts power output based on motion data, offering a computationally efficient way to assess sites for future implant optimization. These findings pave the way for the development of self-powered intracardiac implants (e.g., ICLPs), potentially eliminating the need for high-risk battery replacement surgeries and improving patient outcomes.

To further validate these findings and move toward clinical translation, our next step involves placing the sensor endocardially using a percutaneous, closed-chest approach. This will allow us to assess fluid-structure interactions (between blood flow and capsule) and effects from papillary muscles. Future studies will also involve multiple animals to assess inter-subject variability. Finally, to bridge the translational gap between our animal model and human applications, we plan to use non-invasive cine magnetic resonance imaging in human subjects. This will allow us to reconstruct and quantitatively compare human cardiac motion profiles against the fundamental patterns identified in this study, helping to validate whether our relative findings translate to human physiology.

## Electronic supplementary material

Below is the link to the electronic supplementary material.

Supplementary file1 (PDF 2262 kb).

## Data Availability

Data are available on request.

## Appendix

The material properties of the piezoelectric layer (PZT-5H) are as follows:

Elasticity matrix:$$\left[ {c^{E} } \right] = \left[ {\begin{array}{*{20}c} {127.2} & {80.2} & {84.7} & 0 & 0 & 0 \\ {80.2} & {127.2} & {84.7} & 0 & 0 & 0 \\ {84.7} & {84.7} & {117.4} & 0 & 0 & 0 \\ 0 & 0 & 0 & {23.0} & 0 & 0 \\ 0 & 0 & 0 & 0 & {23.0} & 0 \\ 0 & 0 & 0 & 0 & 0 & {23.47} \\ \end{array} } \right] GPa$$

Coupling matrix:$$\left[ e \right] = \left[ {\begin{array}{*{20}c} 0 & 0 & 0 & 0 & {17.03} & 0 \\ 0 & 0 & 0 & {17.03} & 0 & 0 \\ { - 6.62} & { - 6.62} & {23.24} & 0 & 0 & 0 \\ \end{array} } \right]C/m^{2}$$

Permittivity:$$\left[ \varepsilon \right] = \left[ {\begin{array}{*{20}c} {1704.4} & 0 & 0 \\ 0 & {1704.4} & 0 \\ 0 & 0 & {1433.6} \\ \end{array} } \right]\varepsilon_{0} ,$$ $$\varepsilon_{0} = 8.854 \times 10^{ - 12} F/m$$
