# Supplementary material for: In-Vivo 6D Heart Motion Analysis for Emerging Self-Powered Cardiac Implants
Source: Ann Biomed Eng. 2026 Feb 19;54(7):2237–52. doi: 10.1007/s10439-026-04037-5 (PMC13290797; doi:10.1007/s10439-026-04037-5)
Supplement: Supplementary file 1 — Supplementary file1 (PDF 2262 kb). [file 10439_2026_4037_MOESM1_ESM.pdf]

Supporting Information

In-vivo 6D heart motion analysis for self-powered intracardiac implants development

Milad Hasani[1], John Huber[2], Benedict Kjærgaard[3,4], Tomas Zaremba[3,5], Alireza Rezania[1*], Sam Riahi[3,5]

[1] *AAU Energy, Aalborg University, Aalborg, Denmark*

[2] *Department of Engineering Science, University of Oxford, Parks Rd, Oxford, OX1 3PJ United Kingdom*

[3] *Department of Clinical Medicine, Aalborg University, Aalborg, Denmark*

[4] *Department of Cardiothoracic Surgery, Aalborg University Hospital, Aalborg, Denmark*

[5] *Department of Cardiology, Aalborg University Hospital, Aalborg, Denmark*

S1. BNO055 fusion mode sensor characteristics

Table S1 outlines the key operating limits, bandwidths, resolutions, noise characteristics, and typical offsets of the accelerometer and gyroscope employed in fusion mode. These specifications define the baseline accuracy and dynamic response of the BNO055 sensor.

Table S1- Performance Specifications of accelerometer and gyroscope in Fusion Mode

Parameter	Accelerometer	Gyroscope
Range	±4g	±2000 °/s
Bandwidth	62.5 Hz	32 Hz
Resolution	14-bit	16-bit
Noise Density	~150 µg/√Hz	~0.014 °/s/√Hz
Offset (Typical)	±80 mg	±1 °/s

The noise and filtering relied on both the BNO055 sensor's built-in hardware capabilities and our post-processing software. The sensor itself has built-in frequency filters and an internal fusion algorithm that inherently reduces noise by blending sensor data. In post-processing, we also applied a software low-pass filter to remove any high-frequency noise above 30 Hz. This selection is validated by the spectral analysis in the following Figure, which demonstrates that the physiological signal energy is concentrated below 25 Hz, with a sharp magnitude drop-off at higher frequencies.

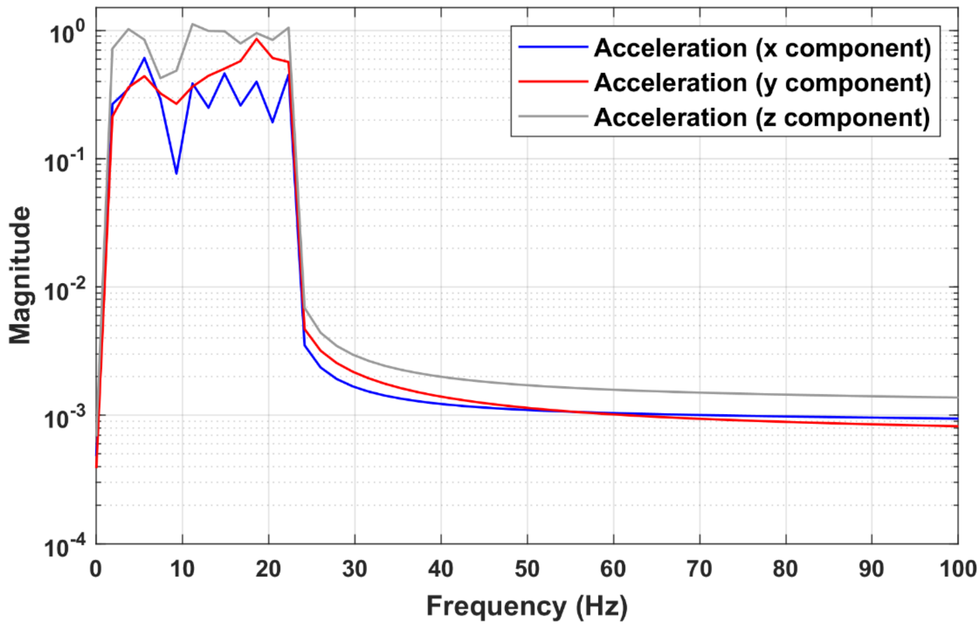

Figure S1- Frequency spectrum (Magnitude) of the acceleration components (x, y, and z) derived from the cardiac motion data. The plot reveals that the physiological signal energy is predominantly confined to the 0–25 Hz bandwidth.

S2. Detailed medical information of in-vivo experimentation

A female Danish Landrace-Yorkshire pig of approximately 50 kg was used for the experiment. Anesthesia was induced with Zoletil 10 ml intramuscular, a mixture of Tiletamine 8.3 mg/ml, Zolazepam 8.3 mg/ml, Butorphanol 1.7 mg/ml, and Xylazine 8.3 mg/ml. After a central venous line was obtained anesthesia was maintained with Propofol and Fentanyl delivered by infusion pumps and adjusted according to the animals' reactions. The animal was intubated for ventilation with a Dräger Primus ventilator (Dräger Medical Deutschland GmbH, Lübeck, Germany). Tidal volume was 10 ml/kg with an end expiratory pressure of 5 kPa; respiratory rate was adjusted according to an end tidal carbon dioxide concentration of 4-6 kPa.

A 6 French arterial catheter was inserted into a femoral artery for continuous blood pressure monitoring and a 10 Fr catheter was put into the left jugular vein for insertion of pacewires. Both catheters were from Cordis Corporation, Florida, USA and were inserted using Seldinger technique. A Foley catheter with a temperature gauge (Covidien, Degania Bet) was inserted into the bladder for urination and monitoring of temperature. To keep temperature constant during the experiment a forced air warming system was used (Mistral-

Air Stryker, Portage, MI, USA). Access to the right jugular vein was done with surgical cut down.

S3. Coordinate system transformation of implanted sensor data

The implanted motion sensor measures the 3D linear acceleration and angular velocity in the moving sensor's body frame (X, Y, Z), which follows the orientation of the sensor, as shown in Figure S1. The Z axis of the sensor's moving body frame is aligned with the normal vector to the local heart surface. However, the operation of proposed energy harvesters relates to inertial force that should be analyzed in the fixed reference coordinate system (x, y, z). Therefore, it is necessary to transform measured data from the body frame to the fixed reference frame. For this coordinate system transformation, the relative 3D rotation of the sensor throughout cardiac cycles should be considered, as shown in Figure S1.

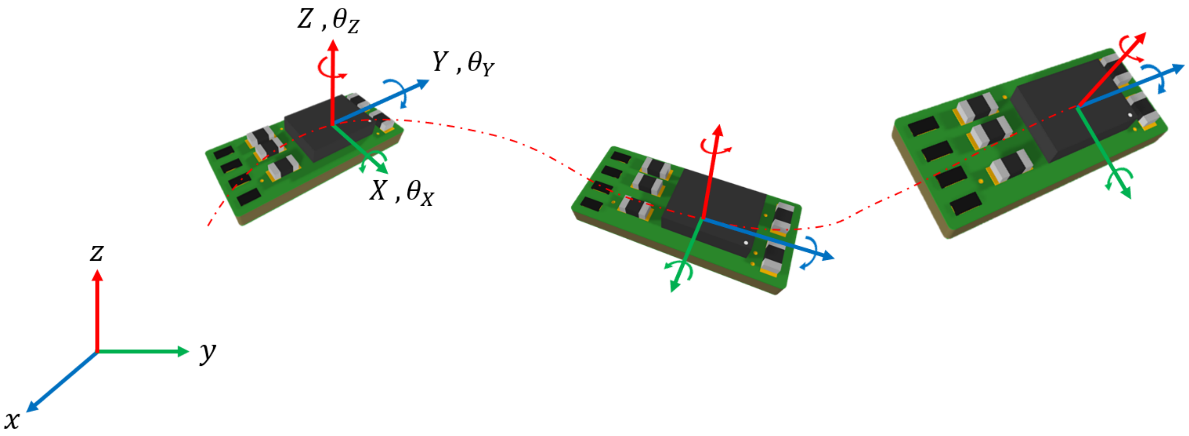

Figure S2- The variable orientation of the sensor's body frame with respect to fixed reference coordinates.

The built-in fusion algorithm provides the real-time orientation based on Euler angles and quaternions. While Euler angles offer an intuitive representation of rotations, they suffer from the well-known issue of gimbal lock. Quaternions provide a robust and efficient representation of rotations by avoiding gimbal lock.

A quaternion consists of four components: one scalar part ($\mathbb{w}$) and three vector components ($\mathbb{x}, \mathbb{y}, \mathbb{z}$). Together, these components define the quaternion as:

$$q = \mathbb{w} + \mathbb{x}i + \mathbb{y}j + \mathbb{z}k \tag{s1}$$

where i, j, and k are unit vectors used to represent the imaginary components of the quaternion. A rotation matrix R can be defined as

$$[R] = \begin{bmatrix} 1 - 2(y^2 + z^2) & 2(xy - wz) & 2(xz + wy) \\ 2(xy + wz) & 1 - 2(x^2 + z^2) & 2(yz - wx) \\ 2(xz - wy) & 2(yz + wx) & 1 - 2(x^2 + y^2) \end{bmatrix} \tag{s2}$$

This rotation matrix enables the transformation of vectors from the body frame $\vec{v} = [v_X, v_Y, v_Z]^T$to the reference frame $\vec{v}' = R.\vec{v}$. Thus, the measured instantaneous linear acceleration vector $\vec{a} = [a_X, a_Y, a_Z]^T$ and angular velocity vector $\vec{\Omega} = [\Omega_X, \Omega_Y, \Omega_Z]^T$ in the body frame are transformed into the fixed reference frame as:

$$\vec{a'}(t) = \begin{bmatrix} a_x \\ a_y \\ a_z \end{bmatrix} = [R] \begin{bmatrix} a_X \\ a_Y \\ a_Z \end{bmatrix} \tag{s3}$$

$$\vec{\Omega'}(t) = \begin{bmatrix} \Omega_x \\ \Omega_y \\ \Omega_z \end{bmatrix} = [R] \begin{bmatrix} \Omega_X \\ \Omega_Y \\ \Omega_Z \end{bmatrix} \tag{s4}$$

Henceforth, all vectors in the fixed reference frame are denoted with a superscript $'$, and the time derivative is shown using a dot. The transformed vectors $\vec{a'}(t)$ and $\vec{\Omega'}(t)$ represent the motion of the BNO055 chip's location that has an offset vector $\vec{r}$ from the heart surface (implant site), as shown in Fig. 3-(a) of the main text. The direction of the vector $\vec{r}$ in the body frame is fixed in the Z direction. The base acceleration $\vec{a'}_{base}$ at the heart surface is recovered from:

$$\vec{a'}_{base}(t) = \vec{a'}(t) - \dot{\vec{\Omega'}}(t) \times \vec{r'}(t) - \vec{\Omega'}(t) \times (\vec{\Omega'}(t) \times \vec{r'}(t)) \tag{s5}$$

In our sensor configuration, the offset vector $\vec{r}$ equals 5 mm. According to Fig. 3 of the main text, while the cardiac motion is measured from the epicardial surface during these animal tests, this data can be employed as an approximation of endocardial motion to analyze endocardial energy harvesters.

Physiological motion over the heart encompasses both respiratory and heartbeat movements [3]. This research concentrates solely on heartbeat motion, so the respiratory motion is filtered out to isolate heartbeat motion. Therefore, the linear acceleration and rotational velocity vectors at the implant site are approximately periodic and can be described by:

$$\vec{a'}_{base}(t) = \vec{a'}_{base}(t + T) \tag{s6}$$

$$\overrightarrow{\Omega'}(t) = \overrightarrow{\Omega'}(t + T)$$

where T represents the duration of a cardiac cycle. All measured cases in this study are examined to derive both time-dependent vectors $\overrightarrow{a'}_{base}(t)$ and $\overrightarrow{\Omega'}(t)$.

S4. Analysis of kinematic criteria variability and statistical distribution

To perform the post-processing, the raw data from all recorded individual cardiac cycles was first loaded. We then established a set of 10-bpm heart-rate (HR) bins (e.g., 75-85 bpm, 85-95 bpm), and each individual cycle was programmatically assigned to one of these bins based on its measured heart rate. For each of the 12 kinematic criteria, the full collection of data points from all cycles within a given bin was analyzed statistically.

The observed variability within each bin stems from a combination of factors: the inherent physiological cycle-to-cycle variation (as no two heartbeats are mechanically identical), the superimposed motion from the respiratory cycle (as breathing motion can affect the measured heart motion), the 10-bpm bin width itself (which groups data from a range of heart rates), and minor sensor measurement noise. This statistical distribution was characterized using the box-and-whisker plots shown in the supplementary material (Section S1). In these plots, the central red line indicates the median value, while the "box" itself represents the interquartile range (IQR). The notches on the box visually represent the 95% confidence interval for the median. The "whiskers" extend to the most extreme data points not considered outliers, and individual outliers are plotted as separate red '+' symbols. This approach allowed us to robustly quantify this combined variability and compare it against the median performance.

- **Implant site 1 (Mid-Septum right):**

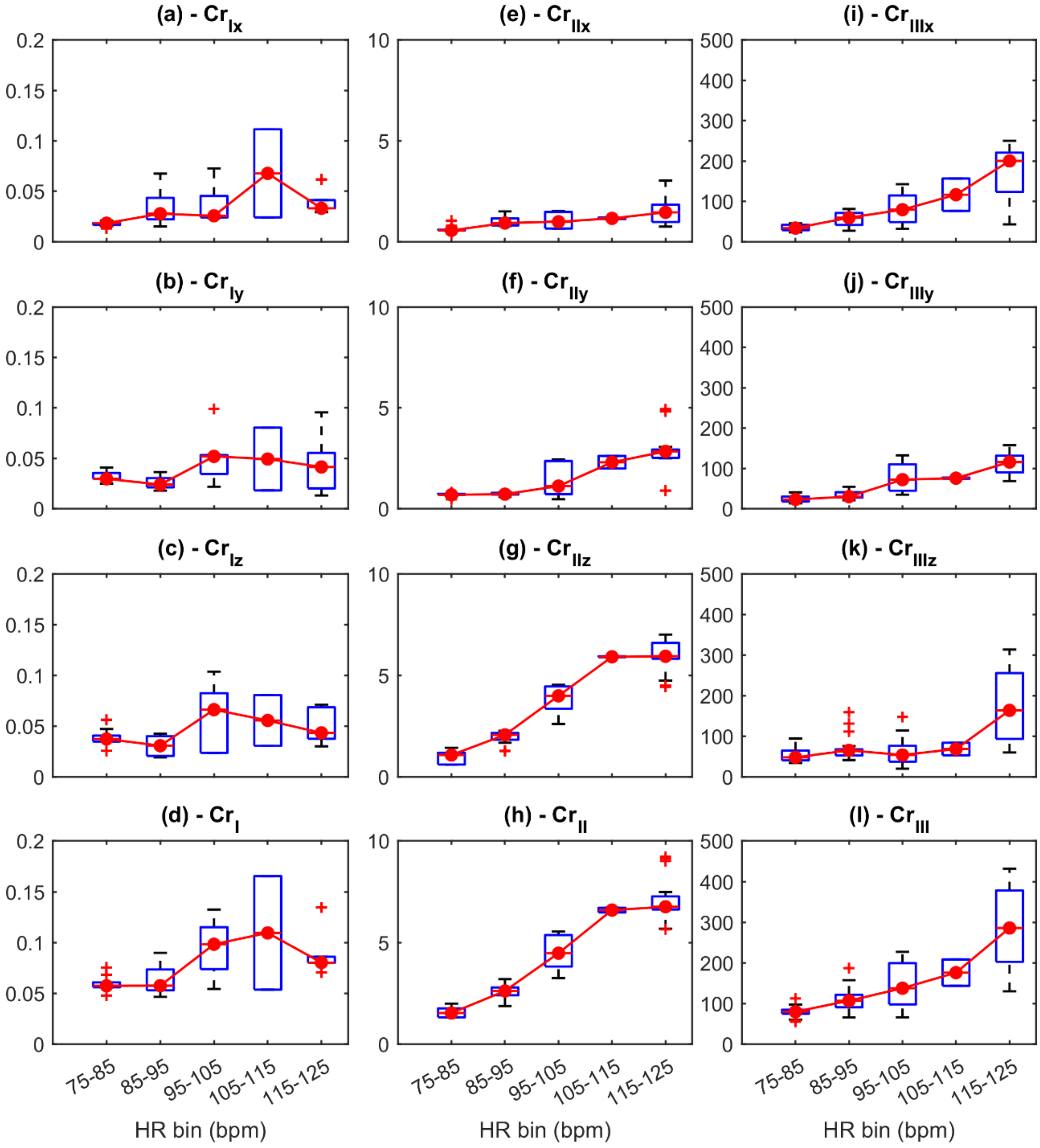

*The figures in the first, second, and third columns are plotted in SI units: m/s, m/s^2, and m/s^3, respectively.

Figure S3- The criteria values at the implant site 1 (mid-septum right); kinetic energy criteria: (a) Cr_{Ix}, (b) Cr_{Iy}, (c) Cr_{Iz}, (d) Cr_{I}, acceleration criteria: (e) Cr_{IIx}, (f) Cr_{IIy}, (g) Cr_{IIz}, (h) Cr_{II}, and jerk criteria: (i) Cr_{IIIx}, (j) Cr_{IIIy}, (k) Cr_{IIIz}, (l) Cr_{III}.

- **Implant site 2 (Right ventricle – outflow tract):**

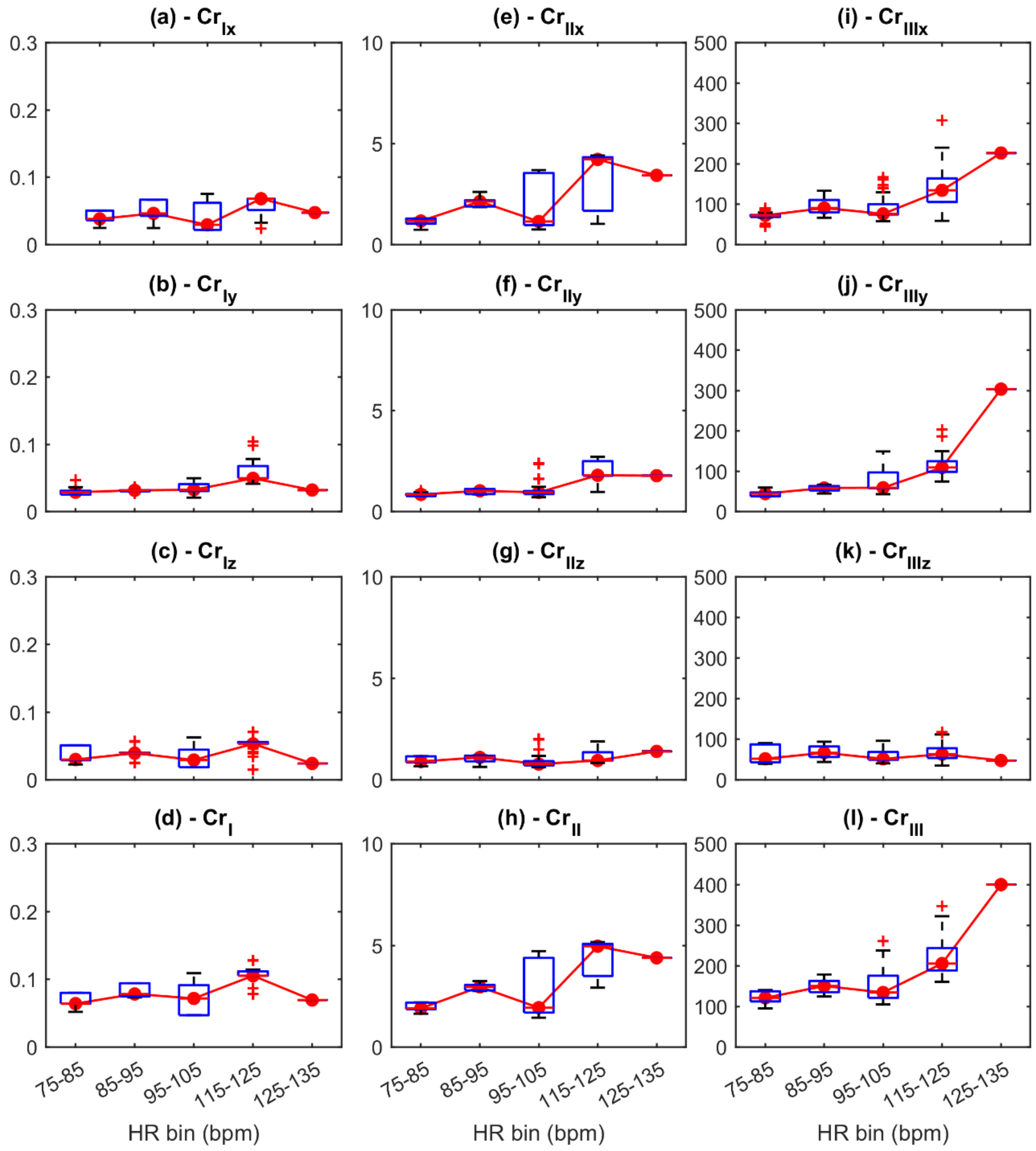

*The figures in the first, second, and third columns are plotted in SI units: m/s, m/s^2, and m/s^3, respectively.

Figure S4- The criteria values at the implant site 2 (right ventricle – outflow tract); kinetic energy criteria: (a) Cr_{Ix}, (b) Cr_{Iy}, (c) Cr_{Iz}, (d) Cr_{I}, acceleration criteria: (e) Cr_{IIx}, (f) Cr_{IIy}, (g) Cr_{IIz}, (h) Cr_{II}, and jerk criteria: (i) Cr_{IIIx}, (j) Cr_{IIIy}, (k) Cr_{IIIz}, (l) Cr_{III}.

- **Implant site 3 (Basal lateral):**

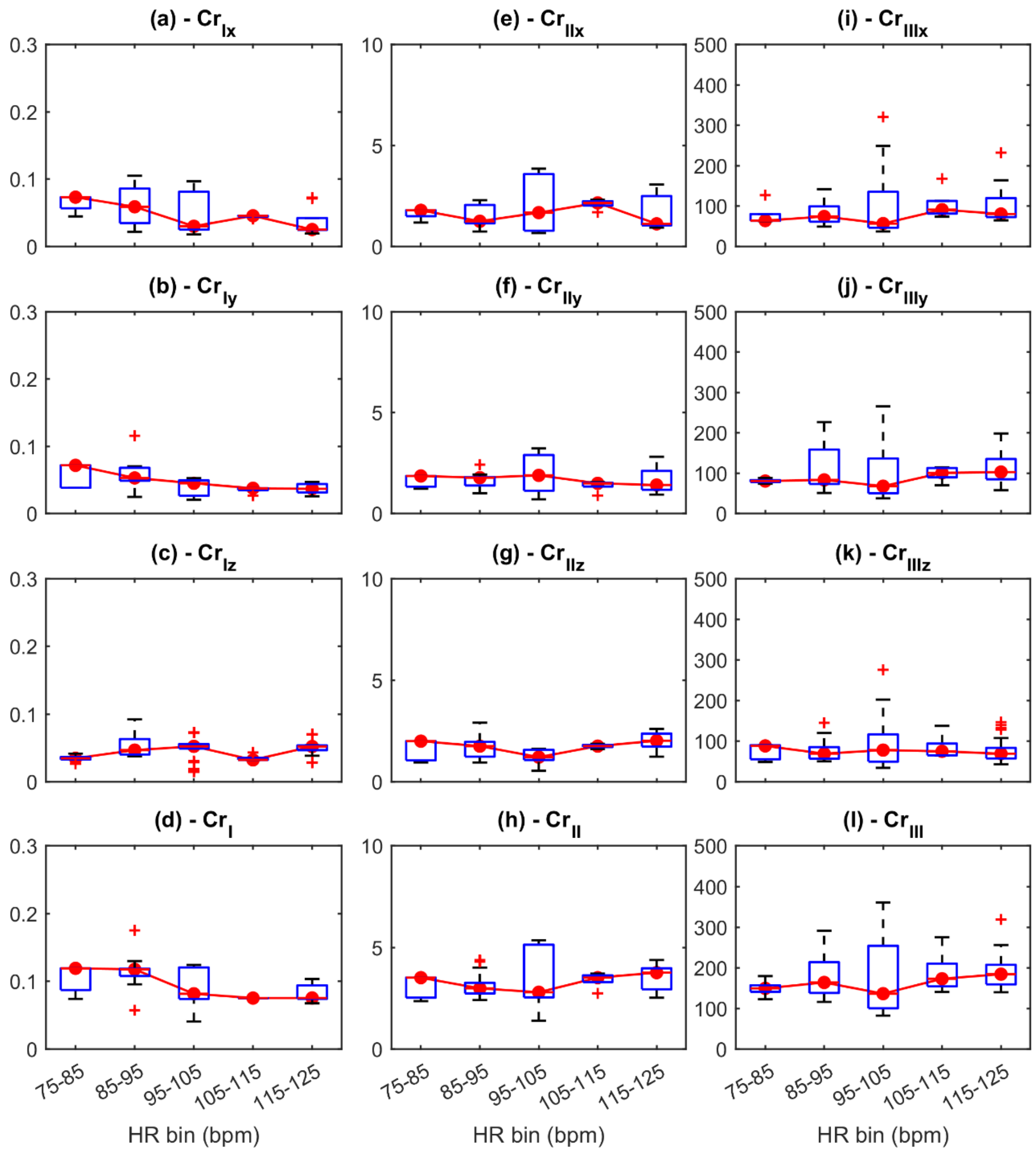

*The figures in the first, second, and third columns are plotted in SI units: m/s, m/s^2, and m/s^3, respectively.

Figure S5- The criteria values at the implant site 3 (basal lateral); kinetic energy criteria: (a) Cr_{Ix}, (b) Cr_{Iy}, (c) Cr_{Iz}, (d) Cr_{I}, acceleration criteria: (e) Cr_{IIx}, (f) Cr_{IIy}, (g) Cr_{IIz}, (h) Cr_{II}, and jerk criteria: (i) Cr_{IIIx}, (j) Cr_{IIIy}, (k) Cr_{IIIz}, (l) Cr_{III}.

- **Implant site 4 (High septum):**

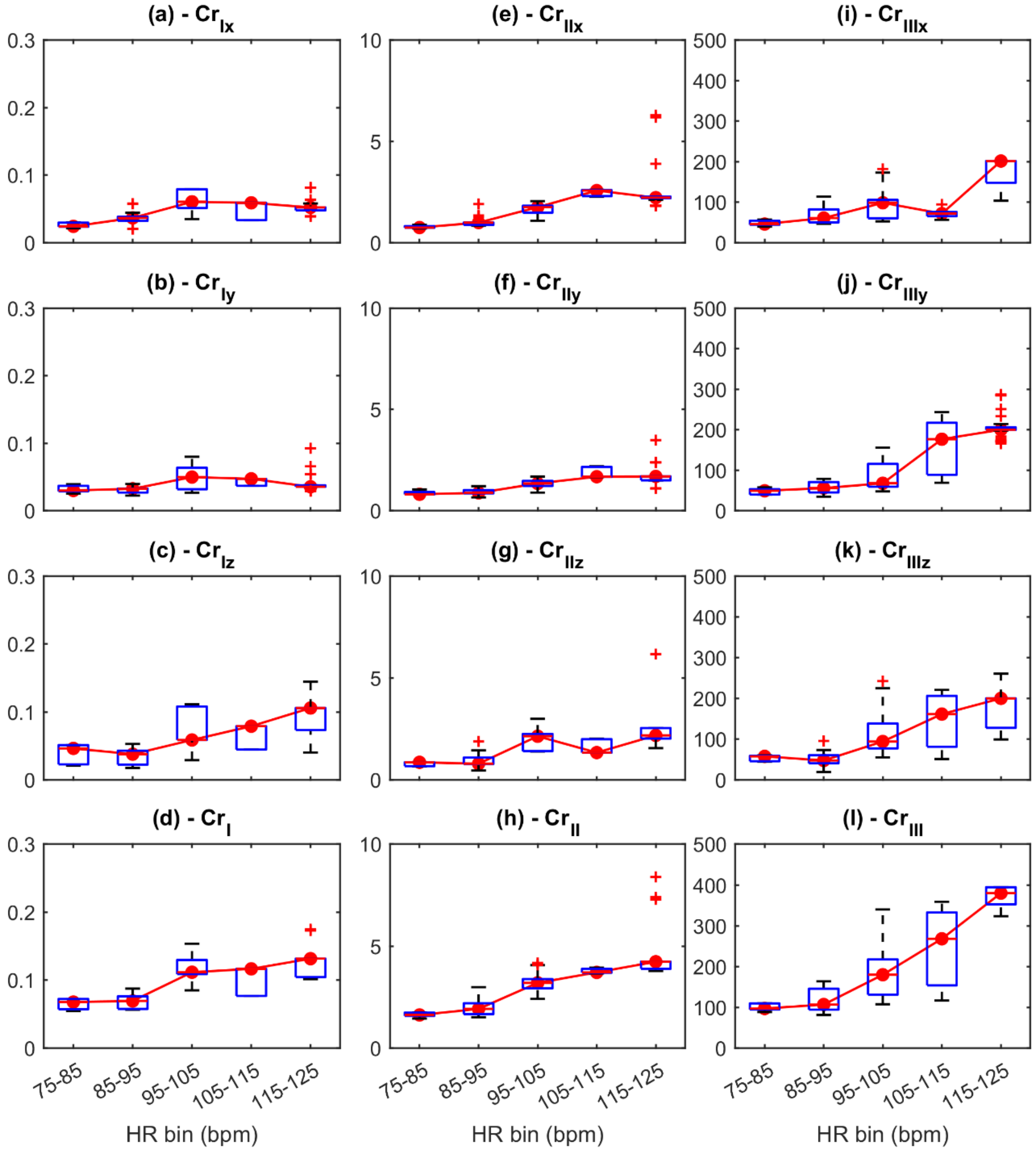

*The figures in the first, second, and third columns are plotted in SI units: m/s, m/s^2, and m/s^3, respectively.

Figure S6- The criteria values at the implant site 4 (high septum); kinetic energy criteria: (a) Cr_{Ix}, (b) Cr_{Iy}, (c) Cr_{Iz}, (d) Cr_I, acceleration criteria: (e) Cr_{IIx}, (f) Cr_{IIy}, (g) Cr_{IIz}, (h) Cr_{II}, and jerk criteria: (i) Cr_{IIIx}, (j) Cr_{IIIy}, (k) Cr_{IIIz}, (l) Cr_{III}.

- **Implant site 5 (Mid-anterior right ventricle):**

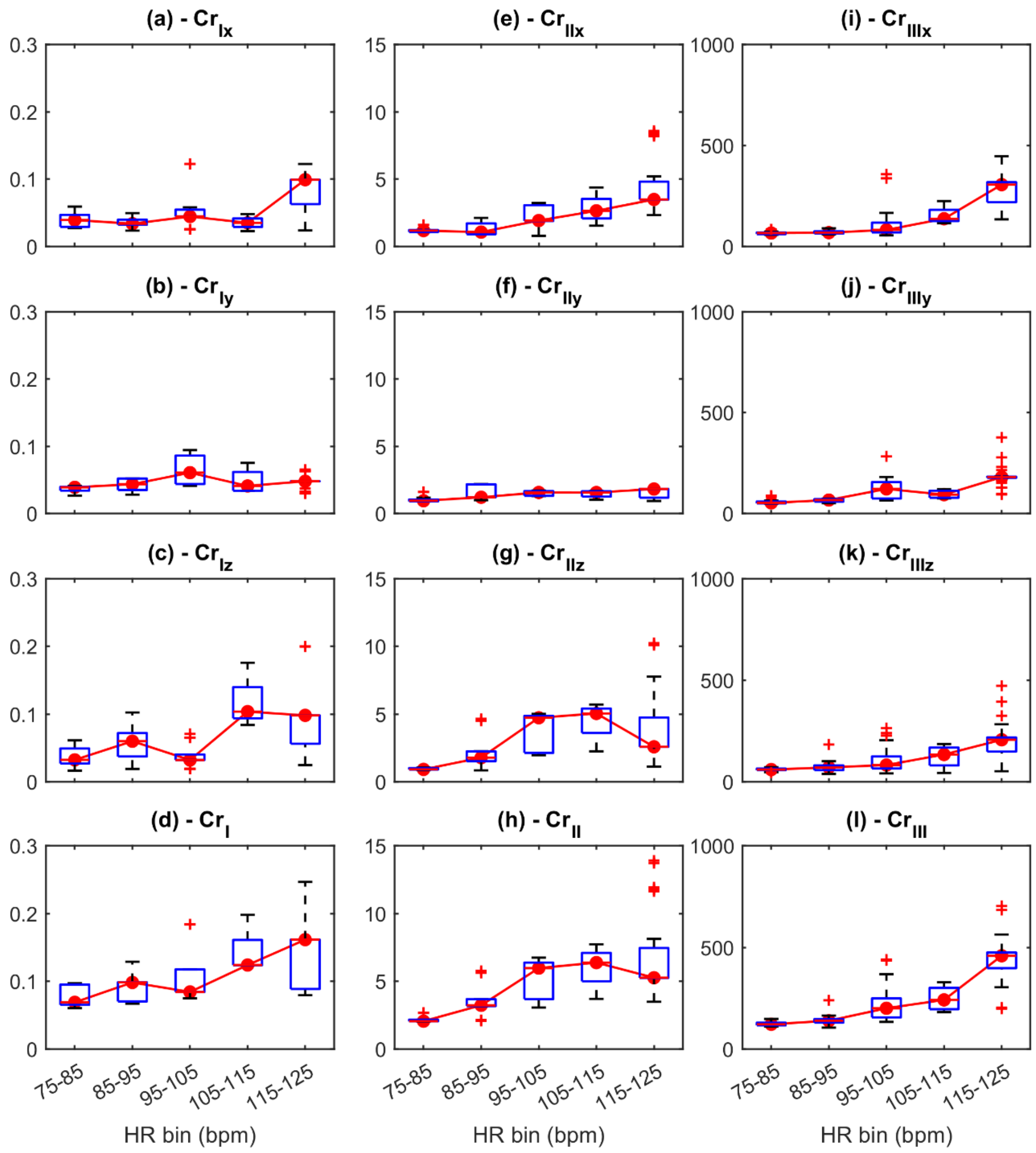

*The figures in the first, second, and third columns are plotted in SI units: m/s, m/s^2, and m/s^3, respectively.

Figure S7- The criteria values at the implant site 5 (mid-anterior right ventricle); kinetic energy criteria: (a) Cr_{Ix}, (b) Cr_{Iy}, (c) Cr_{Iz}, (d) Cr_{I}, acceleration criteria: (e) Cr_{IIx}, (f) Cr_{IIy}, (g) Cr_{IIz}, (h) Cr_{II}, and jerk criteria: (i) Cr_{IIIx}, (j) Cr_{IIIy}, (k) Cr_{IIIz}, (l) Cr_{III}.

- **Implant site 6 (Apex, left ventricle):**

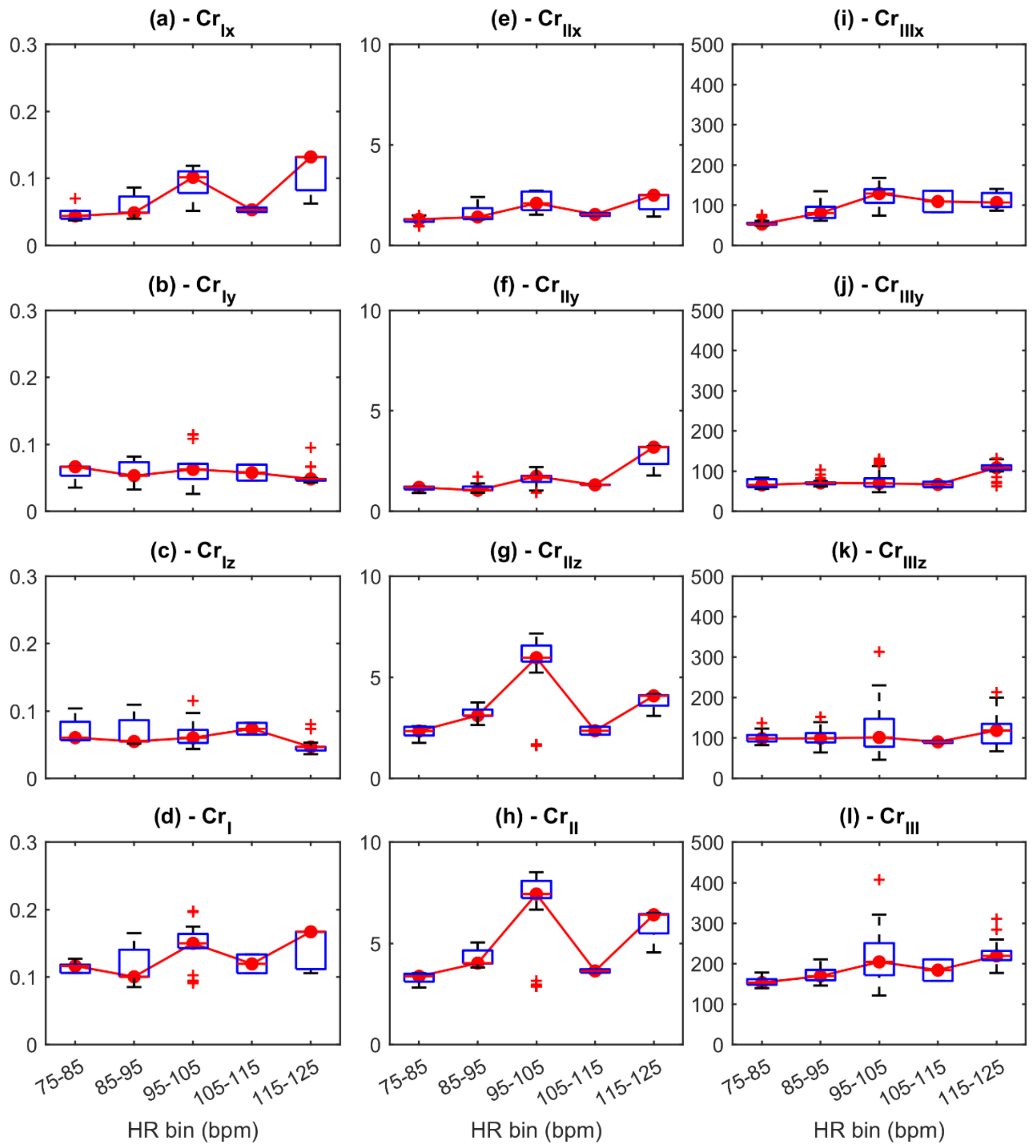

*The figures in the first, second, and third columns are plotted in SI units: m/s, m/s^2, and m/s^3, respectively.

Figure S8- The criteria values at the implant site 6 (apex, left ventricle); kinetic energy criteria: (a) Cr_{Ix}, (b) Cr_{Iy}, (c) Cr_{Iz}, (d) Cr_{I}, acceleration criteria: (e) Cr_{IIx}, (f) Cr_{IIy}, (g) Cr_{IIz}, (h) Cr_{II}, and jerk criteria: (i) Cr_{IIIx}, (j) Cr_{IIIy}, (k) Cr_{IIIz}, (l) Cr_{III}.

While the main manuscript (Table 3) utilizes a normalized scoring system to rank implant sites, the underlying quantitative measurements are provided here for transparency and detailed reference. The following table presents the absolute values of the kinematic criteria calculated for each implant position across five distinct heart rate ranges.

Table S2- Absolute values of the kinematic criteria (Cr_I: Velocity, Cr_{II}: Acceleration, Cr_{III}: Jerk) measured at different implant sites across varying heart rate ranges.

Implant sites	Criteria values at different heart rate ranges														
	75-85 (BPM)			85-95 (BPM)			95-105 (BPM)			105-115 (BPM)			115-125 (BPM)		
	Cr_I	Cr_{II}	Cr_{III}	Cr_I	Cr_{II}	Cr_{III}	Cr_I	Cr_{II}	Cr_{III}	Cr_I	Cr_{II}	Cr_{III}	Cr_I	Cr_{II}	Cr_{III}
Pos 1	0.064	1.91	121	0.078	2.96	150	0.072	1.94	134	0.088	3.45	170	0.105	4.96	205
Pos 2	0.119	3.51	149	0.118	2.99	164	0.082	2.80	136	0.075	3.51	173	0.075	3.76	184
Pos 3	0.068	1.62	97	0.069	1.92	107	0.111	3.20	180	0.116	3.71	268	0.131	4.23	380
Pos 4	0.069	2.06	122	0.098	3.23	140	0.084	5.98	201	0.124	6.38	242	0.161	5.26	459
Pos 5	0.117	3.37	153	0.100	4.02	169	0.150	7.44	204	0.120	3.64	184	0.167	6.41	219
Pos 6	0.064	1.91	121	0.078	2.96	150	0.072	1.94	134	0.089	3.45	170	0.105	4.96	205

* Pos 1: Mid-Septum right, Pos 2: Right ventricle – outflow tract, Pos 3: Basal lateral, Pos 4: High septum, Pos 5: Mid-anterior right ventricle, Pos 6: Apex, left ventricle.

S5. The effect of rotational motion in power generation

The considered energy harvester consists of 25 piezoelectric beams. Figure S2 presents the instantaneous power generation of beam 15 with and without considering rotational motion at a specific motion measurement case. The results indicate that rotational motion can affect the energy harvesting level of each piezoelectric beam.

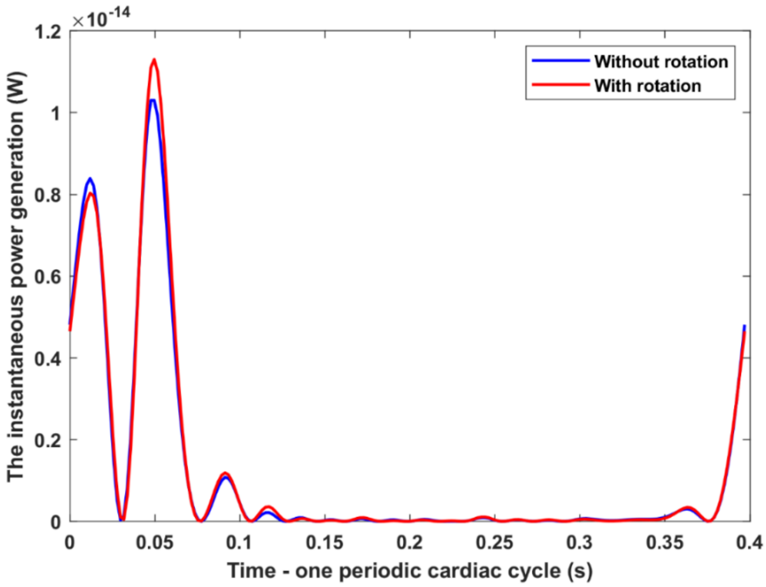

Figure S9- The comparison of the instantaneous power generated by the single piezoelectric beam 15 with and without considering rotational motion at a specific motion measurement case.

S6. Statistical validation of implant site rankings

To validate the energy harvesting potential rankings presented in the main text (Table 4), a rigorous statistical analysis was performed using non-parametric tests, as the kinematic data did not follow a normal distribution. The Kruskal-Wallis H-test was used to determine overall differences between sites, followed by Bonferroni-corrected post-hoc pairwise comparisons to identify specific site superiorities. Statistical significance was defined as $p<0.05$, with high significance at $p < 0.01$, and very high significance at $p < 0.001$.

The following tables present the pairwise comparison results between the optimal site (Left Ventricular Apex, Pos 6) and all other observed epicardial locations for each kinematic criterion: Velocity (Cr_I), Acceleration (Cr_{II}), and Jerk (Cr_{III}).

- Statistical Comparison for Velocity (Cr_I):

The overall comparison across all six implant sites was performed using the Kruskal-Wallis test, which revealed a highly significant global difference in Velocity (Cr_I) distributions ($p = 4.44\times10^{-55}$), rejecting the null hypothesis that the sites are equivalent.

Table S3- Statistical comparison of Velocity (Cr_I) between the Left Ventricular Apex (Pos 6) and other implant sites using Bonferroni-corrected post-hoc analysis.

Comparison	Site A	Site B	p-value	Significance Level
Apex vs. Mid-Septum	Pos 6	Pos 1	1.18×10^{-45}	Significantly Higher
Apex vs. Outflow Tract	Pos 6	Pos 2	9.53×10^{-38}	Significantly Higher
Apex vs. Basal Lateral	Pos 6	Pos 3	3.29×10^{-16}	Significantly Higher
Apex vs. High Septum	Pos 6	Pos 4	2.69×10^{-8}	Significantly Higher
Apex vs. Anterior RV	Pos 6	Pos 5	1.82×10^{-11}	Significantly Higher

For velocity-based metrics, the Left Ventricular Apex is significantly superior to all other tested locations.

- Statistical Comparison for Acceleration (Cr_{II}):

Similarly, the Kruskal-Wallis analysis for Acceleration (Cr_{II}) indicated a statistically significant global difference across the implant sites ($p = 1.70\times10^{-31}$), confirming that inertial

forces vary distinctively depending on the anatomical location.

Table S4- Statistical comparison of Acceleration (Cr_{II}) between the Left Ventricular Apex (Pos 6) and other implant sites using Bonferroni-corrected post-hoc analysis.

Comparison	Site A	Site B	p-value	Significance Level
Apex vs. Mid-Septum	Pos 6	Pos 1	2.31×10^{-18}	Significantly Higher
Apex vs. Outflow Tract	Pos 6	Pos 2	1.45×10^{-25}	Significantly Higher
Apex vs. Basal Lateral	Pos 6	Pos 3	2.43×10^{-13}	Significantly Higher
Apex vs. High Septum	Pos 6	Pos 4	8.07×10^{-16}	Significantly Higher
Apex vs. Anterior RV	Pos 6	Pos 5	0.0014	Significantly Higher

The Apex experiences significantly higher inertial forces (acceleration) compared to all other sites, confirming it as the optimal location for inertial harvesting.

- Statistical Comparison for Jerk (Cr_{III}):

For Normal Jerk (Cr_{III}), the Kruskal-Wallis test also demonstrated a highly significant variation across the epicardium ($p=6.84\times10^{-22}$), although the post-hoc distribution (shown below) indicates a wider spread of high-energy sites compared to velocity or acceleration.

Table S5- Statistical comparison of Normal Jerk (Cr_{III}) between the Left Ventricular Apex (Pos 6) and other implant sites using Bonferroni-corrected post-hoc analysis.

Comparison	Site A	Site B	p-value	Significance Level
Apex vs. Mid-Septum	Pos 6	Pos 1	3.84×10^{-17}	Significantly Higher
Apex vs. Outflow Tract	Pos 6	Pos 2	6.90×10^{-7}	Significantly Higher
Apex vs. Basal Lateral	Pos 6	Pos 3	0.18	Comparable
Apex vs. High Septum	Pos 6	Pos 4	1	Comparable
Apex vs. Anterior RV	Pos 6	Pos 5	1	Comparable

While the Apex is superior to low-motion areas (Pos 1 and 2), the "snap" energy (Jerk) is distributed across the active ventricle. The Apex, High Septum, and Anterior RV offer statistically comparable performance for jerk-based harvesting.

These statistical results validate the rankings presented in Table 4 of the main manuscript. The sites identified as "Rank 1" (Pos 6) exhibit statistically confirmed superiority in Velocity and Acceleration. For Jerk, the clustering of high scores for Positions 4, 5, and 6 in Table 4 is supported by the lack of statistical difference between these groups in Table S4.

- Statistical Comparison for power level of proposed energy harvester:

As established in Eq. 16, the instantaneous power of proposed energy harvester design (shown in Fig. 4) is directly proportional to the square of the lateral jerk magnitude. Therefore, to statistically evaluate the differences in power generation potential, the Kruskal-Wallis test was applied to the power-correlated metric, the square of the normal jerk (Cr_{IIIz}^2).

The statistical analysis confirmed a highly significant variation in theoretical power output potential across the epicardium ($p = 1.31\times10^{-32}$). Post-hoc comparisons revealed that the estimated power potential at the Left Ventricular Apex (Pos 6) was significantly higher than at the Right Ventricle Outflow Tract ($p<10^{-25}$) and Mid-Septum ($p<10^{-14}$). However, no statistically significant difference in power potential was observed between the Apex and the High Septum ($p = 0.128$), suggesting that the High Septum represents a viable alternative site for high-power generation.
